# Specificity in the commonalities of inhibition control: using meta-analysis and regression analysis to identify the key brain regions in psychiatric disorders

**DOI:** 10.1192/j.eurpsy.2024.1785

**Published:** 2024-10-14

**Authors:** Li Wan, Pingting Pei, Qinghui Zhang, Wenxiang Gao

**Affiliations:** 1 Affiliated Psychological Hospital of Anhui Medical University; Anhui Mental Health Center; Hefei Fourth People’s Hospital, Hefei, China; 2 School of Mental Health and Psychological Sciences, Anhui Medical University, Hefei, China; 3 Key Laboratory of Philosophy and Social Science of Anhui Province on Adolescent Mental Health and Crisis Intelligence Intervention, Hefei Normal University, Hefei, China; 4 National Clinical Research Center for Mental Disorders-Anhui Branch, Hefei, China; 5 Department of Psychology, Anhui University, Hefei, China

**Keywords:** ALE meta-analysis, clustering center, factor analysis, inhibition control, psychiatric disorders

## Abstract

**Background:**

The differential diagnosis of psychiatric disorders is relatively challenging for several reasons. In this context, we believe that task-based magnetic resonance imaging (MRI) can serve as a tool for differential diagnosis. The aim of this study was to explore the commonalities in brain activities among individuals with psychiatric disorders and to identify the key brain regions that can distinguish between these disorders.

**Methods:**

The PubMed, MEDLINE, EMBASE, Web of Science, Scopus, PsycINFO, and Google Scholar databases were searched for whole-brain functional MRI studies that compared psychiatric patients and normal controls. The psychiatric disorders included schizophrenia (SCZ), bipolar disorder (BD), major depressive disorder (MDD), obsessive–compulsive disorder, attention-deficit/hyperactivity disorder (ADHD), and autism spectrum disorder (ASD). Studies using go–nogo paradigms were selected, we then conducted activation likelihood estimation (ALE) meta-analysis, factor analysis, and regression analysis on these studies subsequently.

**Results:**

A total of 152 studies (108 with patients) were selected and a consistent pattern was found, that is, decreased activities in the same brain regions across six disorders. Factor analysis clustered six disorders into three pairs: SCZ and ASD, MDD and BD, and ADHD and BD. Furthermore, the heterogeneity of SCZ and ASD was located in the left and right thalamus; and the heterogeneity of MDD and BD was located in the thalamus, insula, and superior frontal gyrus.

**Conclusion:**

The results can lead to a new classification method for psychiatric disorders, benefit the differential diagnosis at an early stage, and help to understand the biobasis of psychiatric disorders.

## Introduction

The diagnosis of psychiatric disorders is typically conducted through clinical interviews and psychological assessments based on the Diagnostic and Statistical Manual of Mental Disorders (DSM) and the International Classification of Diseases (ICD). Neuroimaging and laboratory tests usually help physicians rule out the possibility of other diseases and are not included in the diagnostic criteria. Neuroimaging techniques, such as magnetic resonance imaging (MRI), assist physicians in examining abnormalities in brain structure and function. They play a crucial role in precise localization within the brain during neuromodulation and can also predict the progression and prognosis of certain mental disorders. Moreover, they can serve as a standardized tool for measuring multiple disordered entities.

For example, a meta-analysis revealed that gray matter loss occurred in common areas across six major psychiatric diagnoses: schizophrenia (SCZ), bipolar disorder (BD), major depressive disorder (MDD), addiction, obsessive–compulsive disorder (OCD), and anxiety. The common areas included the dorsal anterior cingulate gyrus, right insula, and left insula. All patients exhibited similar alterations in the integrity of the anterior insula and dorsal anterior cingulate network [1]. Another meta-analysis revealed that brain structural abnormalities in patients with MDD, BD, SCZ, and OCD were highly correlated. The studies revealed commonalities in the hippocampus of patients with major psychiatric disorders and overlapping gray matter reductions in the cingulate cores and the insula in patients with MDD, BD, SCZ, or OCD [2, 3]. A potential shared factor explained 42.3 to 88.7% of the brain structural variation in each disorder [1]. A high proportion of brain regions exhibiting independent mutations were identified in each disorder to identify disorder-specific morphometric abnormalities [2].

Previous studies have focused on the commonalities of brain structures across psychiatric disorders. Whether brain activity is consistent under the same task state remains unclear. Response inhibition, the ability to withhold a prepotent response, is a critical cognitive process that is frequently impaired across numerous psychiatric disorders. The go–nogo task has been extensively utilized as a tool to investigate this capacity, offering insights into the neural underpinnings and behavioral manifestations of psychiatric disorders. Research has consistently demonstrated that individuals with attention-deficit/hyperactivity disorder (ADHD) exhibit poorer performance on + tasks than healthy controls, with increased commission errors (failure to inhibit) and decreased reaction times for correct responses. These findings align with the clinical profile of impulsivity and inattention observed in ADHD patients [4]. In patients with OCD, go–nogo tasks have revealed impairments in response inhibition, particularly when the task involves suppressing responses to obsessive-related cues. These findings support the role of dysregulated inhibitory processes in the maintenance of compulsive behaviors [5]. Patients with depression and bipolar disorder have also shown altered go–nogo task performance, indicating potential deficits in cognitive inhibition. However, the results are less consistent than those for other disorders, possibly due to the heterogeneous nature of mood disorders and the influence of the current affective state on cognitive performance [6]. Common findings include increased commission errors (indicative of poorer response inhibition) and slower reaction times for correct responses. These results suggest a possible underlying neural mechanism contributing to the behavioral phenotype of individuals with ASD [7]. Research has consistently demonstrated that individuals with schizophrenia exhibit poorer performance on go–nogo tasks than healthy controls, with increased commission errors (failure to inhibit) and decreased reaction times for correct responses. These findings align with the clinical profile of cognitive impairment observed in schizophrenia patients [8].

The differential diagnosis of psychiatric disorders is relatively challenging for several reasons [9, 10]: many psychiatric symptoms may be similar or overlapping; the etiology of psychiatric disorders may involve genetic, biochemical, environmental, psychological, and other factors, making it complex to determine the cause of the disorder; the same disorder may present different symptoms and courses in different patients; and psychiatric diagnoses often lack clear biological markers, leading to a reliance on physician experience and patient self-reports. In this context, we believe that task-based MRI can serve as a tool for differential diagnosis. The purpose of this study was to identify common brain activities in individuals with psychiatric disorders and to identify the key brain regions that can differentially regulate these two disorders. To achieve these goals, we first selected qualified functional MRI (fMRI) studies and conducted activation likelihood estimation (ALE) meta-analysis. Factor analysis was then used to cluster the disorders. Regression analysis and residuals were used to identify the key brain regions that can differentiate between the two disorders.

## Methods

### Paradigms included

The go–nogo task is a cognitive test that measures an individual’s ability to inhibit inappropriate responses. It is often used to assess impulsivity, attention, and response control. The task involves presenting participants with a series of stimuli (e.g., letters or numbers) and requiring them to respond quickly to certain stimuli (go trials) while refraining from responding to other stimuli (nogo trials).

There are several types of go–nogo tasks, which vary based on the nature of the stimuli and the response requirements [11, 12]: (a) Simple go–nogo task: In this version, participants are presented with a single type of stimulus (e.g., the letter “A”) and are instructed to respond whenever it appears (go trial) but not when any other letter appears (nogo trial). (b) Choice go–nogo task: This version involves multiple types of stimuli (e.g., different letters or numbers) and requires participants to respond to some stimuli (go trials) while ignoring others (nogo trials). This task requires more cognitive effort than does the simple version and is often used to assess attention and response control in healthy adults. (c) Switching go–nogo task: In this version, participants must switch between responding to one stimulus (go trial) and refraining from responding to another stimulus (nogo trial) based on changing rules or contexts. (d) Emotional go–nogo task: This version incorporates emotional stimuli, such as images or words with positive or negative valence, into the task. In general, go–nogo tasks involve measuring accuracy (correct responses and inhibitions) and reaction time (speed of responding) as indicators of performance.

### Inclusion and exclusion criteria

1. The study was published in a peer-reviewed English language journal.
2. The fMRI study included patients diagnosed with ASD, SCZ, MDD, BD, OCD, and/or ADHD, defined using DSM or ICD criteria; chronic or initial psychosis; no transient psychosis; and no multiple psychiatric diagnoses; or the fMRI study included healthy participants only.
3. The fMRI study using go–nogo paradigms; comparisons between patients and matched healthy controls.
4. The fMRI studies with whole-brain analysis and results after only small volume corrections within the region of interest were excluded; studies focused on structural and resting-state imaging were excluded.
5. The fMRI data were acquired at 0.5–4 T.
6. The whole-brain resolution was 4 mm or lower, and the voxel geometry was isotropic or near-isotropic.
7. The results are expressed in a defined volumetric space (e.g., Talairach space or MNI space) and show the regional activation changes revealed by the task.

### Search strategy

According to the specific procedure, we selected the experimental articles, mainly those describing studies using go–nogo tasks in fMRI scans. PubMed, MEDLINE, EMBASE, Web of Science, Scopus, PsycINFO, and Google Scholar were searched for whole-brain fMRI studies that compared patients and normal controls from 1996 to 2023. The keywords used were as follows: [“response inhibition,” “inhibition control,” “global inhibition,” “inhibition,” “go/no go,” “go no go,” “go–nogo” OR “motor inhibition”] AND [“schizophrenia” OR “attention deficit/hyperactivity disorder” OR “autism spectrum disorder” OR “bipolar disorder” OR “major depressive disorder” OR “obsessive–compulsive disorder”] AND [“fMRI” OR “magnetic resonance imaging”].

The reference lists of the articles were checked for relevant research. Some studies included multiple patient groups and we separated them into comparisons for each diagnostic group versus healthy control participant group.

Two raters searched for the articles and read and decided to keep or discard the studies based on the inclusion criteria. The quality and other information for each article were recorded. The following items were recorded for each study: the adequacy of the control group, the validity of the outcome test, the representativeness of the sample, the representativeness of the surrounding environment of the study, and the appropriateness of the statistical analysis. The score for each item ranged from 0 to 2, representing three levels: low, medium, and high. All the articles were scored in the 0–9 range, with a mean ± SD of 5.63 ± 1.87. All the articles followed a normal distribution, and the articles of very low quality (<3) were excluded from the subsequent analysis.

**Figure 1.**
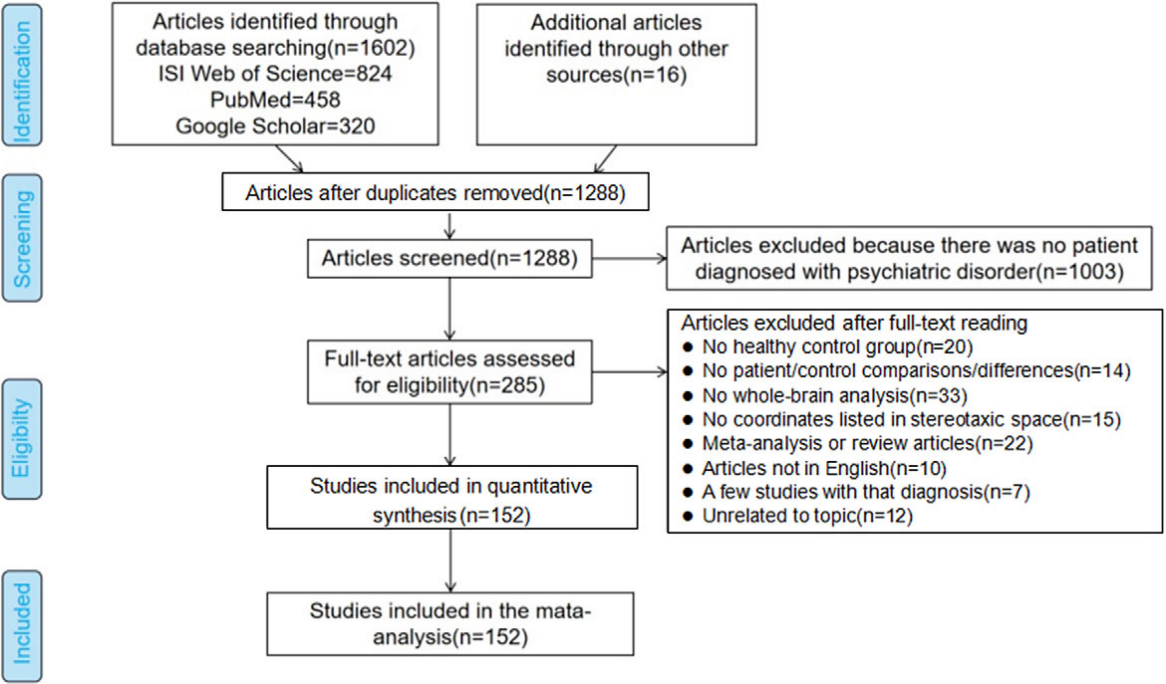
PRISMA flow diagram.

The PRISMA flow diagram is shown in Figure 1 and a detailed description of the included studies is provided in Supplementary Table S1.

### ALE meta-analysis

The use of GingerALE 3.0.2 (www.brainmap.org/ale) allowed us to incorporate variable uncertainties based on the sample size of each study, convert ALE terms from fixed effects to random effects, and add thresholding methods [13]. An ALE meta-analysis of all selected studies collapsing across subject groups (psychiatric disorder vs. normal) was conducted. The included studies compared a psychiatric group with healthy participants and found indicators of regional activity associated with the relevant diagnosis; the peak voxel coordinates from published studies were included in the meta-analysis (see the Supplementary Material for details).

The meta-analysis included a highly diverse sample of diagnoses across several major categories. The sample included six diagnostic groups and 673 comparisons between patients and control individuals from 152 peer-reviewed articles, representing a total of 3,325 patients and 2,140 healthy control individuals. For the analyzed results, six groups of results were compared and a common pattern was obtained.

### Evaluation of robustness

To address the inherent variability in fMRI study designs, protocols, and participant demographics across the included research to ensure the robustness of the meta-analysis findings, we conducted a series of ALE meta-analyses between two subgroups in each group. Based on the median of the factors, we divided the participants into two subgroups: high and low. Factors included voxel, age, stimulus time, stimulus interval, region, and so forth. If both subgroups have cluster centers, comparative analysis can be conducted to further demonstrate the reliability of the analysis results; otherwise, comparative analysis cannot be conducted, and the results may be biased. For details, see the supplementary material.

### Factor analysis and linear regression

In the ALE meta-analysis, the *Z-*score of each brain region was generated and recorded. In statistics, the *Z*-score was defined as a measure that indicates the distance of a value from the mean, expressed in standard deviation units. In a normal distribution, the *Z*-score can be used to determine the position of a value within the distribution. In the ALE meta-analysis, the *Z*-score was used to quantify the activation intensity of brain regions. A high *Z*-score indicated that the activation intensity in that region significantly exceeded the level expected by random chance, suggesting that the area was associated with the cognitive task or behavioral condition being studied. The *Z*-score in the ALE meta-analysis not only provided a method for quantifying the activation strength of brain regions but also, through its standardized nature, enabled comparisons across different studies.

An exploratory factor analysis was used to reduce the dimensionality by taking the *Z*-score of each brain region as an observation and each disorder as a variable. Factor analysis was able to categorize disorders into groups. We then used linear regression analysis to assess the extent to which each disorder was represented by the factor scores of different regions, where the true *Z*-score of each region was predicted by the respective regional factor score. We used a separate linear regression model with the factor scores as independent variables and the raw (true) *Z*-scores as dependent variables. This analysis generated predicted/estimated *Z*-scores and residuals (differences between real and predicted *Z*-scores) for each brain region. The method used in Opel et al.’s (2020) study is described in the Supplementary Material for details.

## Results

### Demographics and variable controls

There were total 152 studies selected, including 16 for SCZ, 14 for OCD, 28 for ADHD, 19 for BD, 16 for MDD, 15 for ASD, and 44 for healthy. The demographic data included age, sex, sample size, and region. Based on the chi-square tests, there was no difference in continent distribution across the six disorders. There were no significant differences in sample size across the six disorders. The participants in the ADHD group were younger than those in the other groups (*p* < 0.05). The proportion of males in the ASD group was greater than that in the healthy, MDD, OCD, and BD groups (*p* < 0.05), and the proportion of males in the SCZ group was greater than that in the MDD group (*p* < 0.05); see Table 1 for details.

**Table 1.** Comparisons among six disorder groups

	Mean ± SD	Group 1	Group 2		Sig.
Age	26.73 ± 9.39			*F* = 5.59	0.000*
		ADHD (18.55 ± 9.24)	Healthy (27.11 ± 4.96)	*t* = −5.83	0.000*
		ADHD	SCZ (33.25 ± 4.33)	*t* = −3.78	0.005*
		ADHD	MDD (31.70 ± 10.27)	*t* = −2.68	0.042*
		ADHD	OCD (34.34 ± 4.21)	*t* = −3.72	0.001*
		ADHD	BD (29.44 ± 11.77)	*t* = −2.75	0.004*
Sex (male/total ratio)	0.63 ± 0.24^a^			*F* = 4.50	0.000*
		ADHD (0.80 ± 0.19)	Healthy (0.58 ± 0.25)	*t* = 4.44	0.000*
		ADHD	MDD (0.39 ± 0.20)	*t* = 4.29	0.006*
		ADHD	OCD (0.55 ± 0.26)	*t* = 2.53	0.018*
		ADHD	BD (0.55 ± 0.10)	*t* = 4.15	0.000*
		ASD (0.84 ± 0.14)	Healthy (0.58 ± 0.25)	*t* = 4.22	0.001*
		ASD	MDD (0.39 ± 0.20)	*t* = 4.37	0.004*
		ASD	OCD (0.55 ± 0.26)	*t* = 2.48	0.032*
		ASD	BD (0.55 ± 0.10)	*t* = 4.82	0.001*
		SCZ (0.76 ± 0.11)	MDD (0.39 ± 0.20)	*t* = 3.60	0.010*
Continent	America, Europe, Asia			χ^2^ = 21.16	0.449
Stimulus type	Color, letter, symbol, picture, word			χ^2^ = 35.40	0.449
Experimental group (*n*)	22.40 ± 20.88			*F* = 0.66	0.723
Control group (*n*)	20.28 ± 18.76			*F* = 0.56	0.784
Total trial	362.27 ± 436.29			*F* = 0.73	0.664
Go/total trial ratio	0.66 ± 0.17			*F* = 0.85	0.558
Stimulation time (s)	1.12 ± 2.38			*F* = 1.13	0.346
Interstimulus interval (s)	2.36 ± 3.55			*F* = 2.44	0.017*
		SCZ (6.45 ± 6.22)	MDD (0.71 ± 0.93)	*t* = 2.02	0.021*
		SCZ	ADHD (1.74 ± 1.07)	*t* = 3.82	0.001*
		SCZ	BD (1.34 ± 1.38)	*t* = 2.55	0.023*
		SCZ	ASD (1.19 ± 0.55)	*t* = 2.24	0.047*

* *p* < 0.05.

a Means 63% ± 24%, a higher value represents more men.

The stimulus types included colors, letters, symbols, pictures, and words. The chi-square test did not show any difference in stimulus type across the six disorders. There was no difference across the six disorders in the total stimulus trials, the go/total trial ratio, or stimulation presentation time. However, the SCZ group had longer interstimulus intervals than did the MDD, ADHD, BD, and ASD groups (*p*-values <0.05); see Table 1.

The ADHD group was younger than the other groups, while there was no significant difference among the other groups. Age was used as a covariate when comparing ADHD patients and controls. The proportion of males in the ASD group was greater than that in the other groups and the proportion of males in the SCZ group was greater than that in the MDD group. The male ratio was used as a covariate in the corresponding comparisons.

Regarding the evaluation of robustness (ALE meta-analysis in subgroups), due to sample size limitations, we selected the following factors for analysis:

1. Voxel as a disturbance factor: (a) It did not cause a difference in the healthy group. Group 1 (voxel ≤ 2 × 2 × 2 mm^3^) had clustering centers, indicating that there were significantly correlated common regions in the experiments within this group; Group 2 (voxel > 2 × 2 × 2 mm^3^) had a clustering center; the presence of clustering centers in the two groups indicated that comparative analysis could be performed, and the reliability of the analysis results was further proven through comparative analysis. (b) In the ADHD group, there was a clustering center in Group 1, but there was no clustering center in Group 2. The results of the two groups were inconsistent, indicating that the significant areas may come from the same group or different groups. That is, voxel size, as a distractor, caused differences in ADHD patients.
2. The stimulation time interval was used as a confounding factor. (a) The stimulation time interval differed in the healthy group. There was a clustering center in stimulation time interval Group 1 (time interval < 2,400 ms) and no cluster center in stimulation time interval Group 2 (time interval > 2,400 ms). (b) There was no difference in the ADHD group. There were cluster centers in stimulation time interval Group 1 (time interval < 2,400 ms) and cluster centers in stimulation time interval Group 2 (time interval > 2,400 ms).
3. As a confounding factor, stimulation time (a) did not cause differences in the healthy group and (b) did not cause differences in the ADHD group.
4. Region (for Europe and North America only) was used as a confounding factor: (a) region did not cause differences in the healthy group and (b) region did cause differences in ADHD incidence.

If two subgroups had cluster centers, comparative analysis could be performed and the reliability of the analytic results was further proven by comparative analysis. If one of the subgroups had no cluster center, a comparative analysis could not be performed. In this case, we removed some extreme values before performing further operations.

Because the sample sizes of the remaining subgroups were too small (*n* < 17), the analysis was of little significance and could not fully represent the overall information. Therefore, the current results partially prove the effectiveness of ALE contrast and more experiments are needed to further confirm the effectiveness of ALE contrast.

### Decreased activities in the same brain regions across 6 disorders

**Figure 2.**
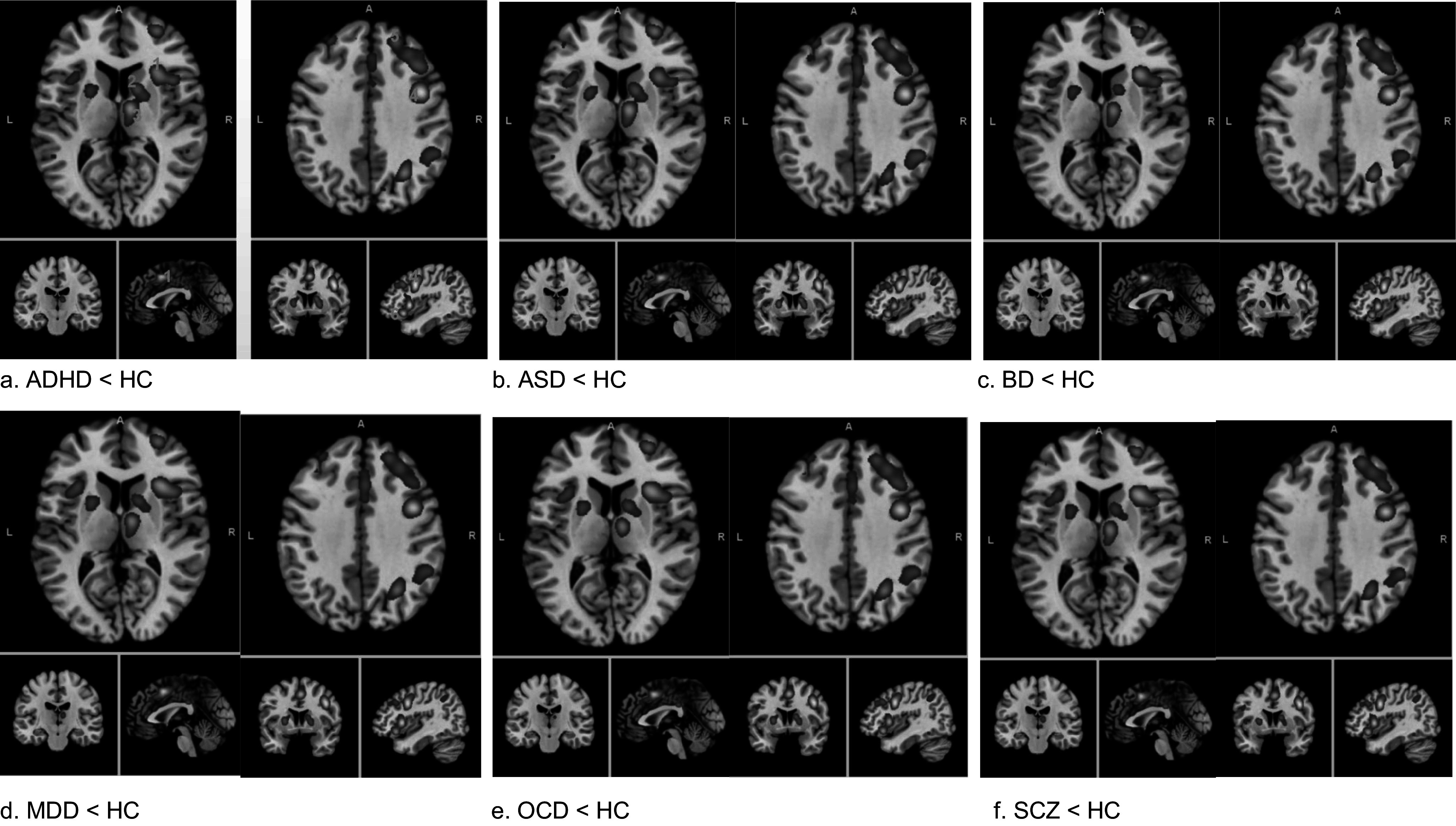
A consistent pattern across six disorders – the decreased activities in patients versus normal controls. The decreased activities were consistently found in the bilateral cingulate gyri, bilateral inferior frontal gyri, bilateral medial frontal gyri, bilateral superior frontal gyri, bilateral precentral gyri, and bilateral insula across all six disorders. Moreover, in the right hemisphere, the decreased activities were consistently found in the right inferior parietal lobule, right superior parietal lobule, and right thalamus in patients. (1. Medial frontal gyrus; 2. Insula; 3. Thalamus; 4. Precentral gyrus; 5. Superior frontal gyrus; 6. Inferior frontal gyrus) (2. A, anterior; L, left; R, right) (
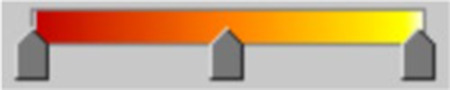
 ALE score range: 0.02–0.07)

Compared to healthy participants, participants with each disorder showed both increased and decreased activity in response to inhibition in different brain regions. However, activities in the bilateral cingulate gyri, bilateral inferior frontal gyri, bilateral medial frontal gyri, bilateral superior frontal gyri, bilateral precentral gyri, and bilateral insula regions consistently decreased in the six disorders. Moreover, in the right hemisphere, activity was consistently decreased in the right inferior parietal lobule, right superior parietal lobule, and right thalamus in patients (five out of six disorders). In contrast, no consistent pattern was found for the regions with increased activity (Figure 2 and Supplementary Tables S2–S4).

### Six disorders dimensionally reduced to three pairs

**Figure 3.**
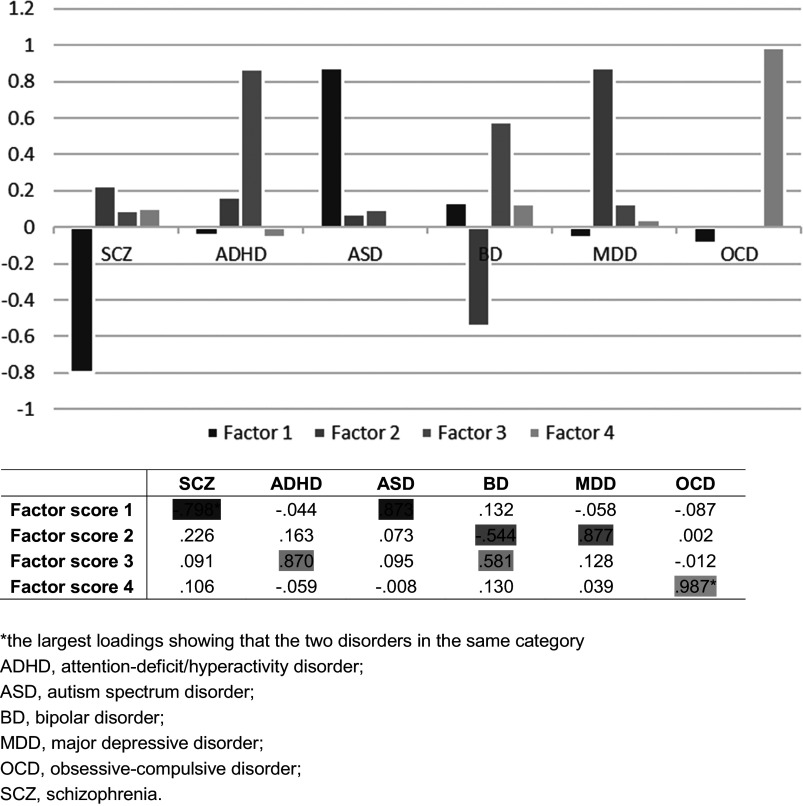
The factor loadings on each disorder. *The largest loadings showing that the two disorders in the same category. ADHD, attention-deficit/hyperactivity disorder; ASD, autism spectrum disorder; BD, bipolar disorder; MDD, major depressive disorder; OCD, obsessive–compulsive disorder; SCZ, schizophrenia.

Factor analysis revealed that SCZ and ASD were mainly associated with factor 1, ADHD and BD were mainly associated with factor 2, MDD and BD were mainly associated with factor 3, and OCD was associated with factor 4. The four extracted factors explained 78.02% of the total variance in the *Z*-scores and explained the information of the original variables well (Figure 3 and Supplementary Table S5).

**Figure 4.**
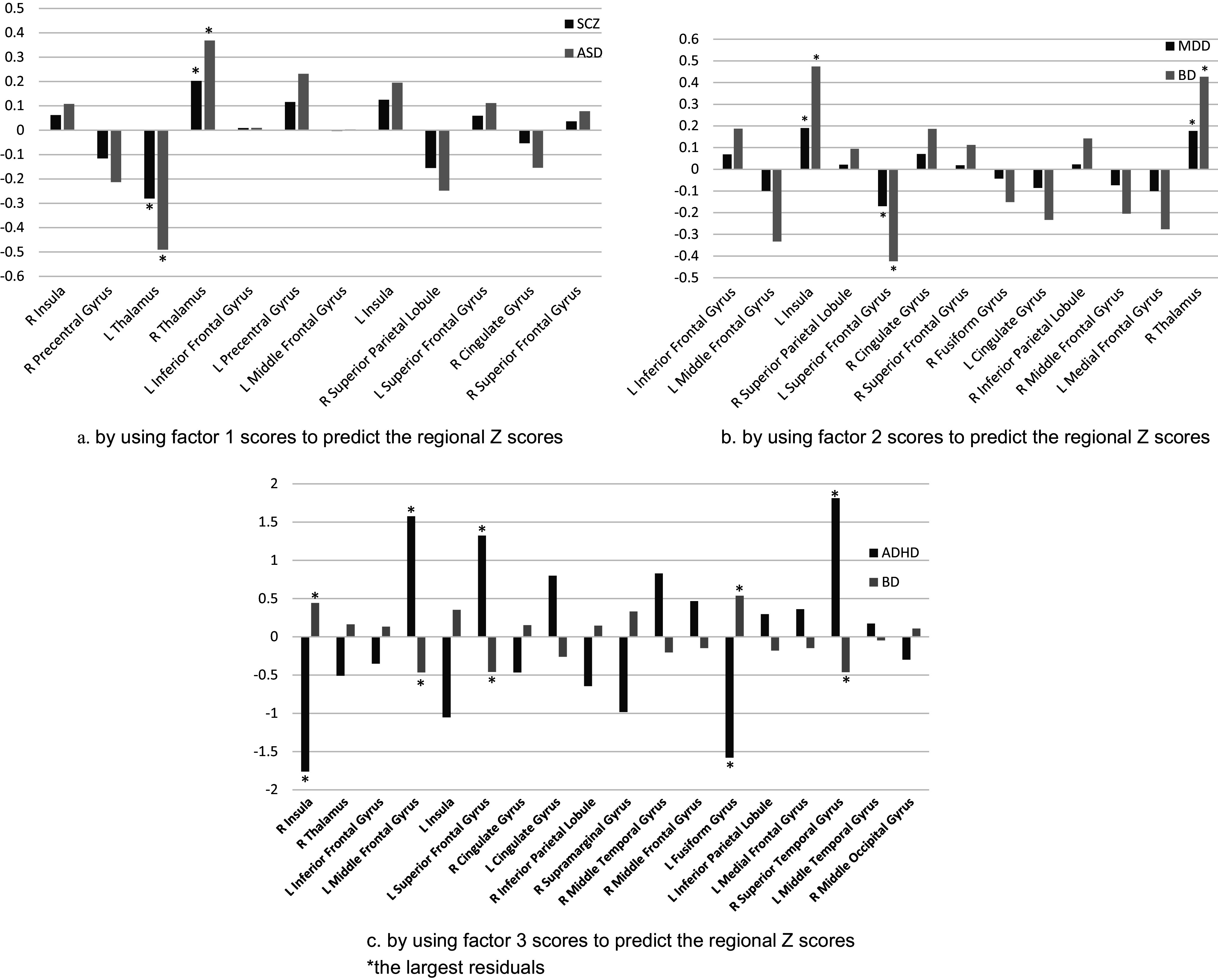
The residual of each brain region in regression analysis. *The largest residuals.

The observation of the rotated component matrix showed that SCZ had a strong negative correlation with factor 1 (≈0.8), and ASD had a strong positive correlation with factor 1 (>0.8). BD had a moderate negative correlation with factor 2 (>0.5), and MDD had a strong positive correlation with factor 2 (>0.8). ADHD had a strong positive correlation with factor 3 (>0.8), and BD had a moderate positive correlation with factor 3 (>0.5). Additional analysis of the relationship between factor scores of each region of interest and their respective original *Z*-scores showed that SCZ and ASD had opposing effects (Figure 4 and Supplementary Table S5).

### Identifying the key brain regions associated with the two disorders

By using factor 1 scores to predict the regional *Z*-scores, we found that the brain regions with the greatest absolute residual values for SCZ patients and ASD patients were the bilateral thalamus, bilateral precentral gyrus, left insula, and right superior parietal lobule. The residual values were at least 50% greater than those of the other samples (Figure 4 and Supplementary Table S6).

By using factor 2 scores to predict the regional *Z*-scores, we found that for ADHD and BD patients, the residuals of the right superior temporal gyrus, right insula, left fusiform gyrus, left superior frontal gyrus, and left middle frontal gyrus were the largest (Figure 4 and Supplementary Table S6).

By using factor 3 scores to predict the regional *Z*-scores, we found that for MDD patients and BD patients, the brain regions with the largest absolute residuals were the left insula, right thalamus, and left superior frontal gyrus (Supplementary Table S6).

## Discussion

This study first provided a comprehensive cross-disorder analysis of brain functional abnormalities related to inhibitory control based on 152 fMRI studies and revealed a consistent pattern in the brain across six disorders. Second, the study reduced the dimensionality of six disorders to three pairs: SCZ and ASD, MDD and BD, and ADHD and BD, which indicated that there was an inherent connection between the two disorders. Third, the regression analysis revealed that the differences between SCZ patients and ASD patients were located in the bilateral thalamus, bilateral precentral gyrus, left insula, and right superior parietal lobule. For MDD and BD patients, the brain regions with the largest absolute residuals were the left insula, right thalamus, and left superior frontal gyrus. For ADHD and BD patients, the residuals of the right superior temporal gyrus, right insula, left fusiform gyrus, left superior frontal gyrus, and left middle frontal gyrus were the largest, indicating that these brain regions are key brain regions that can differentiate disorders.

### Consistent pattern across six disorders

Patients showed consistently decreased activities compared to those of healthy participants in the cingulate gyrus, insula, frontal gyrus (inferior, medial, and superior), and precentral gyrus in both the left and right hemispheres; notably, decreased activities in the precentral gyrus were only present in the right hemisphere in OCD patients. Moreover, in the right hemisphere, decreased activities were consistently found in the right inferior parietal lobule, right superior parietal lobule, and right thalamus in the patient.

Previous studies have identified a brain network engaged in go–nogo tasks that spans the bilateral middle and inferior frontal gyri, mid-cingulate and parietal cortices and the presupplementary motor area [14–16]. These studies pointed to a large lateral frontal cluster spanning Brodmann areas (BAs) 9 and 44–47 common to go–nogo tasks. A meta-analysis also revealed commonly activated regions, namely, the insular cortex and medial BA 6 [1 7]. Another meta-analysis in young healthy subjects identified 12 separate clusters, with major clusters centered in the right insula (BA 13), right middle frontal gyrus (BA 9), right inferior parietal lobule/precuneus (BA 40, 19, 7), and superior frontal gyrus (medial BA 6, 8) [18]. In contrast, our study revealed consistently decreased activities in these brain regions in patients with psychiatric disorders, indicating a dysfunction of these brain regions in psychiatric disorders, and inhibition-related brain activities might be a common neural basis of psychiatric disorders.

### The relationship between SCZ and ASD

The relationships between SCZ patients and ASD patients were grouped into the same category, and the key brain regions in the bilateral thalamus, bilateral precentral gyrus, left insula, and right superior parietal lobule were located. As the largest residual represented a large unpredictability, these regions showed the “same direction but with significant differences” in activity between SCZ patients and ASD patients. We further suggested that these brain regions can differentiate between the two disorders under a task-fMRI condition.

SCZ and ASD were once thought to be the same disorders but in different stages, with ASD manifesting as an early stage of SCZ. Patients with ASD were more likely to have a family history of SCZ and were at increased risk for psychosis [19]. The average incidence of schizophrenic spectrum disorders in the ASD population is 12.8%, whereas the incidence of ASD in individuals with schizophrenic spectrum disorders is approximately 3.6% [1]. The failure to fully understand the psychiatric symptoms and comorbidities among individuals with disorders was a significant barrier to the development of treatment and clinical decisions.

Previous structural MRI studies have consistently revealed decreased gray matter volume and thickness in the insula of patients with SCZ [1, 20]. Patients with ASD exhibit hypoactivity in the insula and thalamus compared with controls during go–nogo tasks [20]. Previous studies have shown that the volumes of areas such as the hippocampus, amygdala, and prefrontal cortex were smaller in SCZ patients than in healthy controls [1]. In contrast, individuals with ASD have larger volumes in areas such as the amygdala and hippocampus, while the prefrontal cortex is smaller [21]. In SCZ patients, there are fewer connections between the prefrontal cortex and other brain regions. In contrast, individuals with ASD have more connections between the prefrontal cortex and other brain regions. There was abnormal metabolic activity in the brain of SCZ patients, especially between the prefrontal cortex and temporal cortex [22]. Abnormal metabolic activity in the brain in individuals with ASD mainly occurs between the amygdala and the hippocampus [23].

Research has demonstrated that individuals with SCZ experience diminished volumes in the hippocampus, amygdala, and prefrontal cortex. These structures play crucial roles in cognitive, emotional, and social functions. For instance, the hippocampus was associated with memory formation, the amygdala with emotional processing, and the prefrontal cortex with decision-making and social behavior [24]. In contrast, those with ASD tend to have enlarged volumes in the amygdala and hippocampus but a decreased volume in the prefrontal cortex. These differences might be related to the cognitive, emotional, and social impairments observed in individuals with ASD. For example, a reduction in the prefrontal cortex could be linked to difficulties in decision-making and social behavior [25].

In conclusion, these brain structural changes offer significant insight into the biological basis of SCZ and ASD. Our study attempted to determine the precise causes of these alterations and how they relate to the development and symptoms of these disorders.

### The relationship between ADHD and BD

ADHD primarily manifests as excessive activity, inattention, emotional instability, and impulsivity. During manic episodes of BD, patients may also exhibit symptoms such as elevated mood, racing thoughts, and increased volition, which are similar to the inattention and emotional instability observed in ADHD patients [26]. In terms of brain function, individuals with ADHD often show deficiencies in executive functions, such as working memory, planning, and organizational abilities, possibly related to abnormalities in the prefrontal cortex. Patients with BD may display different brain functional patterns during various emotional states (mania and depression) [27].

Hypoactivity in the insula and thalamus regions was found in ADHD patients during the go–nogo task [28]. In rapid-response inhibition using the go–nogo task, BD patients exhibit underactive insular and thalamic regions [29]. Previous studies also revealed that patients with BD have smaller volumes in the hippocampus and amygdala than do normal individuals, while the gray matter density in the prefrontal cortex and dorsolateral prefrontal cortex is greater. The brain structure of ADHD patients was characterized by lower gray matter density in the prefrontal cortex and dorsolateral prefrontal cortex but greater gray matter density in the amygdala and basal ganglia. Patients with BD have weaker connections between the prefrontal cortex and other brain regions when performing cognitive tasks; patients with ADHD exhibit strong connectivity between the prefrontal cortex and other brain regions [30].

This study first categorized ADHD and BD patients into the same group in terms of go–nogo-related brain activity and then revealed that the right superior temporal gyrus, right insula, left fusiform gyrus, left superior frontal gyrus, and left middle frontal gyrus exhibited the greatest differences in brain activity in the go–nogo task. In addition, these regions showed opposite directions of brain activity in the two disorders, suggesting a key role in distinguishing the two disorders.

### The relationship between MDD and BD

The depressive episodes in patients with BD and MDD essentially maintain consistent symptoms. The primary distinction between them lies in the presence of mood fluctuations and manic episodes in BD patients. In terms of brain structure and function, extensive research has identified both similarities and differences between them.

Consistent gray matter loss has been found in patients with BD or MDD in the bilateral anterior insula and thalamus [1]. Several meta-analyses revealed reduced gray matter volume in the bilateral insula in patients with MDD and BD [31]. Compared with control patients, MDD patients exhibit increased activation in the anterior insula during successful inhibitory trials [32]. A positive association between depression symptoms and medial thalamus activation was observed during the go–nogo task [33]. The brain structure of patients with MDD is characterized by lower gray matter density in the prefrontal cortex and dorsolateral prefrontal cortex but greater gray matter density in the amygdala and basal ganglia [34]. Patients with BD have weaker connections between the prefrontal cortex and other brain regions when performing cognitive tasks; patients with MDD exhibit stronger connectivity between the prefrontal cortex and other brain regions [31, 35].

Furthermore, the brain regions with the greatest differences between the two groups were the left insula, right thalamus, and left superior frontal gyrus. This finding helps us understand the differences between the two, especially the variations in brain activity under the same cognitive state, and provides a biological basis for distinguishing between the two.

### Conclusion and limitation

In summary, this study is the first to reveal the relationships among six psychiatric disorders through three-step analyze. The six-pair ALE meta-analysis revealed a consistent pattern across six disorders by showing decreased activities in the inhibitory circuitry in the go–nogo task. The factor analysis further clustered the six disorders into three pairs: SCZ and ASD, ADHD and BD, and BD and MDD. The regression analysis ultimately identified the key brain regions that showed different activities (in the same or opposite directions) in the two disorders. The results may lead to a new classification method for psychiatric disorders, benefit the differential diagnosis at an early stage, and help to understand the biobasis of psychiatric disorders. As a supplement to previous studies, this study further confirmed the role of the insula and thalamus in these disorders, providing useful insights for differential diagnosis.

The limitations of the study were as follows: (a) Confounding factors were analyzed in the study, ALE meta-analysis of the subgroups revealed that some subgroups had no cluster center, and a comparative analysis could not be performed. In addition, the sample sizes of the remaining subgroups were too small (*n* < 17), and the analysis was of little significance and could not fully represent the overall information. Therefore, the current results partially prove the effectiveness of ALE contrast, and more experiments are needed to further confirm the effectiveness of ALE contrast. (b) Future studies should focus on developing more standardized go–nogo paradigms, exploring the relationship between task performance and real-world outcomes, and integrating neuroimaging methods to elucidate the neural bases of response inhibition deficits in psychiatric disorders. Longitudinal studies are also needed to assess the predictive value of go–nogo performance for disease progression and treatment response.

Nonetheless, understanding the mechanisms of response inhibition can inform interventions aimed at improving self-control and decision-making abilities in patients. For clinical practice, when performing fMRI scans in the go–nogo paradigm under unified standards, the activation level and direction of key brain regions can be used for the differential diagnosis of two types of disorders. However, to establish standardized diagnostic criteria, a large database is needed. Other cognitive tasks or neurobiological markers can be explored to further understand the commonalities and distinctions in brain function across psychiatric disorders.

## Supporting information

Wan et al. supplementary material Wan et al. supplementary material

## Data Availability

The data are available upon request.

## Abbreviations

ALE: activation likelihood estimation
ADHD: attention deficit hyperactivity disorder
ASD: autism spectrum disorder
BD: bipolar disorder
MRI: magnetic resonance brain imaging
MDD: major depressive disorder
MNI: Montreal Neurological Institute
OCD: obsessive–compulsive disorder
SCZ: schizophrenia

## Supplementary material

The supplementary material for this article can be found at http://doi.org/10.1192/j.eurpsy.2024.1785.
