# Supplementary material for: Specificity in the commonalities of inhibition control: using meta-analysis and regression analysis to identify the key brain regions in psychiatric disorders
Source: Eur Psychiatry. 2024 Oct 14;67(1):e69. doi: 10.1192/j.eurpsy.2024.1785 (PMC11730059; doi:10.1192/j.eurpsy.2024.1785)
Supplement: Wan et al. supplementary material [file S0924933824017851sup001.docx]

**Supplementary**

1. ALE meta-analysis
2. Evaluation of robustness
3. Factor analysis & linear regression

Table S1: General information of the selected studies

Table S2: ALE meta-analysis found decreased activities in each disorder: patients vs. healthy controls

Table S3: ALE meta-analysis found decreased activities in patients in the following brain regions (consistent across disorders)

Table S4: ALE meta-analysis found increased activities in patients in the following brain regions (not consistent across disorders)

Table S5: The factor scores of brain regions in factor analyses

Table S6: Regression analyses on the original scores of SCZ and ASD with the factor 1 scores

1. **ALE meta-analysis**

The activation likelihood estimation (ALE) algorithm has been used to identify consistent patterns in brain activity on fMRI. The algorithm identified the areas that had reported coordinate convergence throughout the experiments^(17)^. For each voxel, the ALE algorithm aggregated the probabilities of all focal points in a given experiment to generate a modeled activation (MA) map. Then, the union of all the modeled activation maps produced a voxel-level ALE score, which reflected the convergence of the results for each specific location in the brain. By comparing the ALE score with the empirical null distribution, the significance of the convergence was evaluated, which reflected the random spatial association between experiments with a fixed within-experiment focus distribution.

The null hypothesis of the ALE method is that there are no overlaps between the MA maps of various experiments in the meta-analysis, and all overlaps are caused by random factors. In the latest algorithm, the MA map of each experiment is arranged according to the MA value of each voxel and transformed into a histogram, thereby obtaining a histogram containing the effective MA value of each voxel but not including the spatial position information of the voxel. Subsequently, the histograms of the MA maps of each experiment are successively combined to obtain the null hypothesis distribution. Specifically, the histograms of two experimental MA maps are first integrated to obtain a combined histogram. Then, this combined histogram is integrated with the histogram of the third experimental MA map, until the histograms of all experimental MA maps are integrated, obtaining the distribution of the null hypothesis. After calculating the cross-experimental MA map and the corresponding null hypothesis distribution map, a significance test can be performed, i.e., testing whether the actual cross-experimental activation probability of each voxel has a significant difference from the activation probability in the corresponding null hypothesis distribution. After the significance test, each voxel will obtain a statistical test p-value. If the p-value is less than a specific threshold, the null hypothesis can be rejected, suggesting that this voxel indeed appears within the brain region represented by at least one activation peak. To overcome the false positive problem brought by multiple comparisons, researchers use strict multiple comparison correction methods, such as false discovery rate (FDR) and familywise error rate (FWE). For voxels that have significant results after strict FDR or FWE method tests, their significance is: in multiple experiments of the meta-analysis, this voxel is stably activated. To obtain significantly activated brain regions, the ALE method identifies voxels that are significant and spatially connected based on each voxel's p-value, i.e., clusters. The ALE method records the statistical indicators of these clusters, including volume, boundaries, center coordinates, and peak ALE values within the region. Significantly activated clusters can be labeled using existing brain atlas toolkits, thereby obtaining the anatomical location of significantly activated brain regions.
 Under the zero distribution of random spatial associations throughout the experiment, the observed ALE score was compared with the expected ALE score. Then, the obtained nonparametric P value was set as a threshold and converted into a Z score to be displayed under the cluster-level family error correction threshold, p < 0.05 (voxel-level P formation threshold p < 0.005). All clusters were set to be at least 200 mm^3^. The focal point of the Talairach space was converted to the MNI space, and all the coordinates reported in the study were in the MNI space.

To determine the common areas activated or inactivated in response to go/no-go tasks, we performed an association analysis. That is, we used the minimum statistics under the conjunctive null hypothesis and calculated the intersection of the threshold meta-analysis graphs derived from these two methods. The difference between activation possibilities was tested by separately performing ALE on experiments related to any group and calculating the voxel differences between subsequent ALE mappings. Then, all the studies with data that were included in the above two analyses were summarized and randomly divided into two groups of the same size as the two original experiments. The activation probability estimation scores of these two groups were calculated to reflect the null hypothesis of label interchangeability, and the difference between these ALE scores for each voxel in the brain was recorded. This process was repeated 10,000 times, and then the three-dimensional vertical distribution of the difference in ALE scores between the 2 (subitems) analyses was obtained. Then, the true difference in the ALE score was tested against the null distribution, and the P value of each voxel difference was obtained based on the ratio.

**2. Evaluation of robustness**

We introduced confounding factors for subgroup analysis. Comparative analysis is the comparison of two ALE datasets; the joint image shows the similarity between the data; the comparison image of the two ALEs is generated directly by subtracting one input image from the other. If there were significant clustering center coordinates in the comparative analysis (comparative analysis was the bidirectional phase), the data were considered to be from different subgroups.

1. Age as a confounding factor

(1) Age in the healthy group could not be analyzed (Note: sample size unbalanced, it is not significant to perform statistical analysis)

(2) Age differed among the ADHD group (number of experiments: 28) (the sample size was not sufficient for comparative analysis).

b. Voxel as a confounding factor

(1) There was no difference in voxels among the healthy group (number of experiments: 44).

There were cluster centers in voxel Group 1 (voxel <= 2x2x2mm^3^) (number of experiments: 20), indicating that there were significantly correlated common regions in this group. There were clustering centers in voxel Group 2 (voxel > 2x2x2mm^3^) (number of experiments: 24), indicating that there were significantly correlated common regions in this group of experiments.

(2) Voxels had no difference in ADHD (number of experiments: 60) (comparative analysis could not be performed)

There were cluster centers in voxel Group 1 (voxel <= 2x2x2mm^3^) (number of experiments: 14), indicating that there were significantly correlated common regions in this group. There was no cluster center in voxel Group 2 (voxel > 2x2x2mm^3^) (number of experiments: 46), indicating that there was no significantly correlated area in this group of experiments.

The two results were inconsistent, indicating that the significant area may come from the same group or from different groups. In other words, this result proves that there may be insufficient evidence that ‘the ADHD-related comparisons are not interfered with by voxels’.

c.The stimulation interval as a confounding factor

(1) There was no difference in the stimulation time interval among the healthy group (number of experiments: 44) (comparative analysis could not be performed due to the unbalanced sample sizes).

There were cluster centers in stimulation interval Group 1 (time interval <2400 ms) (number of experiments: 36), indicating that there were significantly correlated common regions among the experiments in this group.There was no cluster center in stimulation interval Group 2 (time interval>2400 ms) (number of experiments: 8), indicating that there was no significantly correlated region in the experiments within this group.

(2) Stimulation intervals were not different between the ADHD group and the control group (number of experiments: 72) (comparison analysis showed that there were significant cluster center coordinates).

There were cluster centers in stimulation interval Group 1 (time interval < 2400ms) (number of experiments: 46), indicating that there was a significantly correlated common region among the experiments in this group. There were cluster centers (number of experiments: 10) in stimulation interval Group 2 (time interval > 2400ms)(number of experiments: 26), indicating that there was a significantly correlated common region among the experiments in this group.

d. Stimulation time as a confounding factor

(1) Stimulation timing did not differ among the healthy groups (number of experiments: 44) (comparison analysis results showed that there were significant cluster center coordinates).

There were cluster centers in stimulation time Group 1 (stimulation time <1200 ms)(number of experiments: 25) , indicating that there was a significantly correlated common region among the experiments in this group. There were cluster centers in stimulation time Group 2 (stimulation time > 1200ms) (number of experiments: 19), indicating that there were significantly correlated common regions in this group of experiments.

e. Areas as a confounding factor

(1) There was no difference between the region and healthy group (comparative analysis could not be performed because the sample size was not large enough).

There were cluster centers in the America (number of experiments: 13), indicating that there were significantly correlated areas in the experiments within this group. There were cluster centers in Europe (number of experiments: 18), indicating that there were significantly correlated areas in each experiment within this group.

(2) There was no difference in ADHD prevalence between regions (comparative analysis could not be performed because the sample sizes were unbalanced).

There was no cluster center in the America (number of experiments: 16), indicating that there was no significantly correlated area in this group of experiments. There were cluster centers in Europe (number of experiments: 45), indicating that there were significantly correlated areas in each experiment within this group.

Overall, if there are cluster centers for both, comparative analysis can be performed, and the reliability of the analysis results can be further proven through comparative analysis; if not, comparative analysis cannot be performed, and the results may be biased. Because the sample sizes of the remaining subgroups were too small (n < 17), the analysis was of little significance and could not fully represent the overall information. The results partially proved that our comparisons are effective.

1. **Factor analysis & linear regression**

Second, factor analysis aimed to use the few factors that comprehensively reflect most of the information of all variables (disorders). The resulting factors were uncorrelated with each other, eliminating multicollinearity. Third, the deterministic part of the prediction model was composed of functions of the independent variables of prediction, which contained all the interpretable and predictable information in the regression model. The difference between the predicted and observed values must therefore be impossible to predict by chance. In other words, there was no interpretable/predictable information in the error. The largest residual therefore represented a large unpredictability^(16)^.

Factor loadings generated by factor analysis showed how shared variance in the brain is clustered across different disorders, expressing the extent to which extracted common factors influenced the original variable. In addition, factor scores for each brain region indicate the degree to which a particular brain region drove that factor. The regional residuals obtained from these regression analyses represented the difference between the real and predicted regional Z scores. The higher the absolute residuals were, the worse the fitting effect of the shared potential factor based on the real Z score was. Thus, a higher absolute residual indicated a lower degree of sharing between a common factor and a disorder.

**Table S1: General information of the selected studies (SCZ, MDD, OCD, ADHD, BD, ASD, Healthy)**

**a.SCZ**

|  | **First Author** | **Number of participants in the experimental group** | **Number of participants in the control group** | **Age (mean)** | **Male to female ratio** | **Country** | **Total number of trials** | **Proportion of Go trials** | **Stimulus type (others 0, color 1, letter 2, symbol 3, picture 4, word 5)** | **Duration of a single stimulus (ms)** | **Interval between two stimuli (ms)** | **Tesla** | **Voxel**（mm） | **Quality score** |
| --- | --- | --- | --- | --- | --- | --- | --- | --- | --- | --- | --- | --- | --- | --- |
| 1 | Rubia | 6 | 7 | 40 |  | UK | 180 | 0.7 | 4 | 1000 | 650 | 1.5T | 1.5x1.5x3 | 7 |
| 2 | Carter | 17 | 16 | 33.5 | 0.7 | USA | 125 | 0.5 | 2 | 500 | 9500 | 1.5T | 3.75x3.75x3.75 | 9 |
| 3 | Laurens | 10 | 16 | 32.6 | 0.8 | Britain |  | 0.8 | 2 | 240 | 2000 | 1.5T | 3.75x3.75x5 | 7 |
| 4 | Holmes | 17 | 9 | 27 |  |  | 160 | 0.7 | 2 | 500 | 9500 | 1.5T | 3.75x3.8 | 7 |
| 5 | Kaladjian | 21 | 21 | 35.3 | 0.9 | France | 100 | 0.5 | 3 | 250 | 16041.5 | 3T | 3x3x3 | 8 |
| 6 | Zandbelt | 24 | 48 | 31.1 | 0.7 | Netherlands | 474 | 0.9 | 3 | 1000 | 1000 |  |  | 6 |
| 7 | Liddle | 93 | 36 | 19.2 | 1.4 | UK | 304 | 0.8 | 2 |  |  |  |  | 5 |
| 8 | Laurens | 22 | 26 | 11 | 1.2 | UK | 300 | 0.8 | 2 |  | 2000 |  |  | 5 |
| 9 | Tikàsz | 24 | 22 | 36 | 1 | Cnada | 288 | 0.8 | 4 | 500 | 700-1300 | 3T | 3.5x3.5x3.5 | 7 |
| 10 | Fortier | 44 | 22 | 34.3 | 1 |  | 288 | 0.8 | 4 | 500 | 700-1300 | 3T | 3.5x3.5x3.5 | 7 |
| 11 | Nishimura | 14 | 40 | 36.1 | 0.4 | Japan | 64 | 0.5 |  | 500 | 2500 | 3T | 3.5x3.5x3.5 | 7 |
| 12 | Reif | 195 | 72 | 44 | 0.5 | Germany | 400 | 0.1 | 2 | 200 | 1650 |  |  | 6 |
| 13 | Frederike | 21 | 19 | 39.6 | 0.8 | Germany | 145 | 0.8 | 0 | 800 | 2.200-5.200 | 3T | 3x3x5 | 7 |
| 14 | Arce | 17 | 17 | 40.88 |  | USA | 180 | 0.7 | 3 | 1500 |  |  |  | 5 |
| 15 | Fryer | 23 | 72 | 22.51 | 0.7 | USA | 412 | 0.8 | 2 | 250 | 1675 | 3T | 3.44x3.44x5 | 7 |
| 16 | Ford | 11 | 11 | 38 | 0.7 | USA | 330 | 0.9 | 2 | 100 | 1000-3000 |  |  | 6 |

**b.MDD**

|  | **First Author** | **Number of participants in the experimental group** | **Number of participants in the control group** | **Age (mean)** | **Male to female ratio** | **Country** | **Total number of trials** | **Proportion of Go trials** | **Stimulus type (others 0, color 1, letter 2, symbol 3, picture 4, word 5)** | **Duration of a single stimulus (ms)** | **Interval between two stimuli (ms)** | **Tesla** | **Voxel**（mm） | **Quality score** |
| --- | --- | --- | --- | --- | --- | --- | --- | --- | --- | --- | --- | --- | --- | --- |
| 1 | Elliott | 10 | 10 | 42.2 | 0.3 | UK | 240 | 0.5 | 5 | 300 | 900 | 2T | 3x3x3 | 9 |
| 2 | Langenecker | 20 | 22 | 37.6 | 0.7 | USA | 1244 | 0.2 | 2 | 500 | 0 | 3T | 3.75x3.75x4 | 7 |
| 3 | Taylor | 25 | 15 | 35.2 | 0.3 | UK | 245 | 0.8 | 1 | 10004 | 2253 | 3T |  | 7 |
| 4 | Matthews | 15 | 16 | 27.5 | 0.2 | USA | 288 | 0.8 | 2 | 1300 | 200 | 3T | 1x1x1 | 7 |
| 5 | Yang | 13 | 13 | 16 | 0.5 | USA | 288 | 0.8 | 2 | 1300 | 200 | 3T | 0.98x0.98x1 | 7 |
| 6 | Victor | 16 | 14 | 13.5 | 0.8 | USA |  |  | 2 | 26000 | 2000 | 3T | 3.75x3.75x7 | 7 |
| 7 | Stange | 43 | 33 | 21.4 | 0.3 | USA |  |  | 4 |  |  | 3T |  | 3 |
| 8 | Jenkins | 31 | 44 | 21.1 | 0.3 | USA |  |  | 0 | 500 | 2000 | 3T |  | 4 |
| 9 | Rao | 20 | 51 |  |  | USA | 420 | 0.1 | 2 | 500 | 0 | 3T | 3.75x3.75x4 | 4 |
| 10 | Piani | 12 | 12 | 66.5 | 0.6 | Italy |  | 0.5 | 0 |  | 1000 | 3T | 1x1x1 | 6 |
| 11 | Crane | 47 | 54 | 33.6 | 0.3 | USA |  |  | 2 | 500 | 0 | 3T |  | 5 |
| 12 | Stange | 57 | 33 | 21.4 | 0.35 | USA |  |  | 2 |  | 500 | 3T |  | 5 |
| 13 | Malejko | 28 | 27 | 15.79 | 0.22 | Switzerland | 264 | 0.5 | 0 | 200 | 1700 | 3T | 1x1x1 | 6 |
| 14 | Chuang | 106 | 34 | 15.61 | 0.77 | UK |  |  | 5 | 450 | 750 | 3T | 3x3x3 | 5 |
| 15 | Richard | 23 | 26 | 41.3 | 0.4 | France | 144 | 0.5 | 2 | 500 | 700-1300 | 3T | 1x1x1 | 5 |
| 16 | Colich | 15 | 15 | 15.61 | 0.83 | USA | 240 | 0.7 | 3 | 750 | 3000-9000 | 3T |  | 5 |

**c.OCD**

|  | **First Author** | **Number of participants in the experimental group** | **Number of participants in the control group** | **Age (mean)** | **Male to female ratio** | **Country** | **Total number of trials** | **Proportion of Go trials** | **Stimulus type (others 0, color 1, letter 2, symbol 3, picture 4, word 5)** | **Duration of a single stimulus (ms)** | **Interval between two stimuli (ms)** | **Tesla** | **Voxel**（mm） | **Quality score** |
| --- | --- | --- | --- | --- | --- | --- | --- | --- | --- | --- | --- | --- | --- | --- |
| 1 | Elliott | 10 | 10 | 42.2 | 0.3 | UK | 240 | 0.5 | 5 | 300 | 900 | 2T | 3x3x3 | 9 |
| 2 | Langenecker | 20 | 22 | 37.6 | 0.7 | USA | 1244 | 0.2 | 2 | 500 | 0 | 3T | 3.75x3.75x4 | 7 |
| 3 | Taylor | 25 | 15 | 35.2 | 0.3 | UK | 245 | 0.8 | 1 | 10004 | 2253 | 3T |  | 7 |
| 4 | Matthews | 15 | 16 | 27.5 | 0.2 | USA | 288 | 0.8 | 2 | 1300 | 200 | 3T | 1x1x1 | 7 |
| 5 | Yang | 13 | 13 | 16 | 0.5 | USA | 288 | 0.8 | 2 | 1300 | 200 | 3T | 0.98x0.98x1 | 7 |
| 6 | Roth | 12 | 14 | 37.8 | 0.7 | LBN | 216 | 0.5 | 3 | 200 | 2000-4000 | 1.5T | 2x2x2 | 7 |
| 7 | Maltby | 14 | 14 | 39.4 | 0.6 | USA | 100 | 0.8 | 2 | 50 | 1000-3000 | 1.5T | 4x4x4 | 7 |
| 8 | Masharipov | 14 | 34 | 27.1 | 0.4 | Switzerland | 600 | 0.3 | 4 | 100 | 2800-3200 | 3T | 3x3x3 | 9 |
| 9 | Becker | 113 |  | 13.1 | 0.6 | USA |  | 0.75 | 2 | 500 | 3500 |  |  | 4 |
| 10 | Tolin | 48 | 24 | 33.5 | 0.5 | USA |  | 0.85 | 0 | 50 | 1000 |  |  | 2 |
| 11 | Wang | 46 | 26 | 26.5 | 0.4 | China |  |  | 2 |  |  |  | 1.5 × 4.3 × 2 | 0 |
| 12 | Berlin | 9 | 10 | 33.3 | 0.6 | USA | 576 | 0.75 | 4 | 1000 | 1500 |  |  | 7 |
| 13 | Page | 10 | 11 | 39.1 | 1.0 | UK | 208 | 0.88 | 3 | 300 | 1800 |  |  | 7 |
| 14 | Hagland | 29 |  | 30.9 | 0.4 | Norway |  |  | 3 |  |  | 3T | 3x3x3 | 0 |

**d.ADHD**

|  | **First Author** | **Number of participants in the experimental group** | **Number of participants in the control group** | **Age (mean)** | **Male to female ratio** | **Country** | **Total number of trials** | **Proportion of Go trials** | **Stimulus type (others 0, color 1, letter 2, symbol 3, picture 4, word 5)** | **Duration of a single stimulus (ms)** | **Interval between two stimuli (ms)** | **Tesla** | **Voxel**（mm） | **Quality score** |
| --- | --- | --- | --- | --- | --- | --- | --- | --- | --- | --- | --- | --- | --- | --- |
| 1 | Lukito | 69 | 22 | 23 | 1 | UK | 300 | 0.7 | 3 | 1000 | 700 |  |  | 0 |
| 2 | Durston | 7 | 7 | 8.6 | 0.9 |  | 285 | 0.8 | 4 | 500 | 3500 | 1.5T | 3.12x3.12x4 | 7 |
| 3 | Durston | 11 | 22 | 14.5 | 1 | Netherlands | 285 | 0.8 | 4 | 500 | 3500 | 1.5T | 4x4x4 | 4 |
| 4 | Mulder | 24 | 12 | 14.7 | 1 | Netherlands | 360 | 0.7 | 4 | 3000 | 4000 | 1.5T | 4x4x4 | 4 |
| 5 | Suskauer | 25 | 25 | 10.5 | 0.6 | USA | 254 | 0.8 | 1 | 300 | 1500 | 1.5T | 3.59x3.59x4.5 | 7 |
| 6 | Dibbets | 16 | 13 | 28.9 |  | Netherlands | 175 | 0.9 | 2 | 500 | 4000 | 1.5T | 1x1x1 | 6 |
| 7 | Cubillo | 11 | 14 | 29 |  | UK | 156 | 0.8 | 3 | 250 | 1800 | 1.5T | 1.5x1.5x3 | 7 |
| 8 | Hoekzema | 19 | 3 | 11.2 | 0.8 | Spain | 108 | 0.5 | 3 | 1400 | 600 | 1.5T | 0.86x0.86x1.4 | 6 |
| 9 | Schneider | 19 | 17 | 33 | 0.6 | Germany | 432 | 0.5 | 2 | 420 |  | 1.5T | 3.75x3.75x5 | 8 |
| 10 | Kooistra | 10 | 10 | 21.5 | 1 | Canada | 205 | 0.8 | 3 | 500 | 1500 | 3T |  | 4 |
| 11 | Passarotti | 26 | 15 | 14 | 0.5 | USA | 180 | 0.5 | 4 | 800 | 350 | 3T | 3x3x3 | 9 |
| 12 | Dillo | 15 | 15 | 33.5 | 0.7 | Germany | 360 | 0.5 | 2 | 800 | 200 | 1.5T | 2x2x2 | 9 |
| 13 | Rubia | 12 | 13 | 12.5 | 1 | UK | 156 | 0.8 | 3 |  | 1800 | 1.5T | 3.1x3.1x7 | 5 |
| 14 | Mulligan | 12 | 12 | 30.8 | 1 | USA | 1500 | 0.8 | 2 | 1000 | 500 | 1.5T |  | 7 |
| 15 | Braet | 20 | 38 | 13.7 | 0.8 | Ireland | 450 | 0.9 | 0 | 313 | 1439 | 3T | 0.9x0.9x0.9 | 6 |
| 16 | Sebastian | 20 | 24 | 31.8 | 0.6 | Germany | 300 | 0.7 | 2 | 500 | 500 | 3T | 1x1x1 | 6 |
| 17 | Siniatchkin | 17 | 14 | 10 | 0.8 | Germany | 300 | 0.9 | 1 | 300 | 1500 | 3T | 3x3x3 | 6 |
| 18 | Carmona | 19 | 19 | 31.4 |  | Spain | 225 | 0.7 | 2 | 250 | 1500 | 1.5T |  | 6 |
| 19 | Hart | 30 | 30 | 14 | 1 | UK | 296 | 0.8 | 3 | 1000 | 1800 | 3T | 1.87x1.87x3 | 5 |
| 20 | Wang | 28 | 31 | 9.5 |  | China | 500 | 0.1 | 0 | 300 | 1200 | 1.5T | 3.75x3.75x6 | 6 |
| 21 | Cubillo | 19 | 29 | 13.5 | 1 | UK | 296 | 0.8 | 3 | 500 | 1800 | 3T | 3x3x5.5 | 5 |
| 22 | Congdon | 35 | 62 | 35.5 | 0.5 | USA | 128 | 0.8 | 3 | 1000 | 2250 |  |  | 7 |
| 23 | Vasic | 14 | 12 | 25 | 1 | Germany | 264 | 0.5 | 2 | 200 | 1700 | 3T | 3.6x3.6x3 | 6 |
| 24 | Chantiluke | 37 | 25 | 13.5 | 1 | UK | 294 | 0.8 | 3 | 500 | 1300 |  |  | 5 |
| 25 | Janssen | 21 | 17 | 10.6 | 0.9 | USA | 60 | 0.7 | 4 | 1500 | 3000 | 1.5T | 3x3x3 | 7 |
| 26 | van | 185 | 235 | 17.3 | 0.7 | USA | 240 | 0.8 | 4 | 250 |  |  |  | 7 |
| 27 | Ma | 15 | 15 | 9.8 | 0.5 | China | 130 | 0.8 | 2 | 1000 | 1000 | 3T | 2x2x2 | 6 |
| 28 | Spinelli | 13 | 17 | 10.6 | 0.7 | USA | 254 | 0.7 | 1 | 300 | 1500 | 1.5T | 3.5x3.5x3.5 | 7 |

**e.BD**

|  | **First Author** | **Number of participants in the experimental group** | **Number of participants in the control group** | **Age (mean)** | **Male to female ratio** | **Country** | **Total number of trials** | **Proportion of Go trials** | **Stimulus type (others 0, color 1, letter 2, symbol 3, picture 4, word 5)** | **Duration of a single stimulus (ms)** | **Interval between two stimuli (ms)** | **Tesla** | **Voxel**（mm） | **Quality score** |
| --- | --- | --- | --- | --- | --- | --- | --- | --- | --- | --- | --- | --- | --- | --- |
| 1 | Elliott | 8 | 11 | 35.6 | 0.4 | Britain | 60 | 0.5 | 5 | 300 | 900 | 2T | 1x1x1 | 7 |
| 2 | Altshuler | 11 | 13 | 36 | 0.6 | USA | 112 | 0.5 | 2 | 500 | 1500 | 3T |  | 9 |
| 3 | Nelson | 25 | 17 | 14 | 0.5 | USA | 320 | 0.5 | 2 | 250 | 500 | 3T |  | 9 |
| 4 | Wessa | 17 | 17 | 44.9 | 0.6 | France | 144 | 0.7 | 4 | 500 | 1000 | 1.5T |  | 6 |
| 5 | Strakowski | 16 | 16 | 19.5 | 0.7 | USA | 1919 | 0.8 | 1 | 450 | 50 | 4T |  | 7 |
| 6 | Kaladjian | 10 | 10 | 40.8 | 0.5 | France | 294 | 0.8 |  |  |  | 3T | 1x1.2x1.73 | 6 |
| 7 | Kaladjian | 20 | 20 | 36.3 | 0.5 | France | 100 | 0.5 | 2 | 250 | 5000 | 3T | 1x1.2x1.73 | 8 |
| 8 | Welander | 27 | 28 | 34.7 | 0.7 | Norway | 104 | 0.8 | 2 | 500 | 1069 | 1.5T | 2x2x2 | 6 |
| 9 | Singh | 26 | 22 | 15.4 | 0.6 | USA | 144 | 0.5 | 2 | 500 | 1500 | 3T | 3.12x3.12x3.5 | 9 |
| 10 | Pavuluri | 13 | 13 | 14.4 | 0.5 | USA | 180 | 0.5 | 4 | 800 | 350 | 3T | 3x3x3 | 9 |
| 11 | Townsend | 32 | 30 | 37 | 0.6 | USA | 112 | 0.5 | 2 | 500 | 1500 | 3T | 3.12x3.12x3 | 9 |
| 12 | Xiao | 34 | 17 | 15.06 | 0.4 | China | 192 | 0.7 | 4 | 500 | 1000 | 3T | 3x3x3 | 7 |
| 13 | Xiao | 18 | 17 | 15.2 | 0.5 | China | 192 | 0.7 | 4 | 500 | 1000 | 3T | 3x3x3 | 7 |
| 14 | Diler | 10 | 10 | 15.6 | 0.2 | USA | 120 | 0.75 | 2 | 500 | 1000 | 3T | 3x3x3 | 9 |
| 15 | Palermo | 10 | 10 | 46.9 | 0.6 | Italy | 232 | 0.83 | 2 | 250 | 1000 | 1.5T | 1x1x1 | 7 |
| 16 | Penfold | 19 | 20 | 36.3 | 0.47 | USA |  | 0.75 | 2 | 500 | 1500 | 3T | 3.1x3.1x3.0 | 8 |
| 17 | Diler | 12 | 10 | 15.5 | 0.2 | USA | 120 | 0.75 | 2 | 500 | 1000 | 3T | 3x3x3 | 9 |
| 18 | Joshi | 45 | 45 | 39.9 | 0.53 | USA | 112 | 0.50 | 4 | 2000 |  | 3T | 1x1x1 | 7 |
| 19 | Ajilore | 16 | 16 | 43.0 | 0.54 | USA | 112 | 0.50 | 4 | 2000 |  | 3T | 2x2x2 | 7 |

**f.ASD**

|  | **First Author** | **Number of participants in the experimental group** | **Number of participants in the control group** | **Age (mean)** | **Male to female ratio** | **Country** | **Total number of trials** | **Proportion of Go trials** | **Stimulus type (others 0, color 1, letter 2, symbol 3, picture 4, word 5)** | **Duration of a single stimulus (ms)** | **Interval between two stimuli (ms)** | **Tesla** | **Voxel**（mm） | **Quality score** |
| --- | --- | --- | --- | --- | --- | --- | --- | --- | --- | --- | --- | --- | --- | --- |
| 1 | Schmitz | 10 | 12 | 35 | 1 | Britain | 208 | 0.9 | 3 | 500 | 1800 | 1.5T | 0.89x0.89x1.5 | 7 |
| 2 | Kana | 12 | 12 | 24.7 | 0.9 | USA | 180 | 0.8 | 2 | 500 | 500 | 3T | 2x2x2 | 7 |
| 3 | Lee | 12 | 12 | 10.17 | 0.8 | USA | 540 | 0.8 | 2 | 1000 | 1500 | 3T | 2x2x2 | 7 |
| 4 | Duerden | 16 | 17 | 27.5 | 0.7 | Canada | 384 |  | 4 | 500 | 1250 | 3T | 3.28x3.28x3.3 | 6 |
| 5 | Daly | 14 | 14 | 31.4 | 1 | UK | 208 | 0.8 | 3 | 500 | 1800 | 1.5T | 1.87x1.87x3 | 7 |
| 6 | Shafritz | 15 | 15 | 18.1 | 0.8 | USA | 64 | 0.5 | 5 | 500 | 1000 | 3T | 2x2x2 | 7 |
| 7 | Velasquez | 19 | 22 | 25.8 | 0.7 | USA | 576 | 0.8 | 5 | 1000 | 500 | 3T | 1x1x1 | 7 |
| 8 | Goldberg | 11 | 15 | 10.4 | 0.73 | USA | 254 | 0.75 | 0 | 300 | 1500 | 1.5T | 3.59×3.59×4.5 | 9 |
| 9 | Lukito | 21 |  | 22.8 | 1 | UK | 220 | 0.73 | 0 | 300 | 700 | 3T |  | 5 |
| 10 | Langen | 21 | 22 | 25.57 | 1 | Netherlands | 300 | 0.73 | 2 | 1200 | 100 | 3T | 2.4x2.4x2.4 | 6 |
| 11 | Ambrosino | 19 | 19 | 11.5 | 1 | Netherlands | 228 | 0.75 | 0 | 500 | 3500 | 3T | 3x3x3.5 | 6 |
| 12 | Prat | 16 | 17 | 25.3 | 0.6 | USA |  | 0.5 | 0 | 1000 | 500 | 3T | 2x2x2 | 6 |
| 13 | Colich | 18 | 15 | 15.6 | 0.17 | USA | 240 | 0.75 | 4 | 1000 | 2000-6000 | 3T |  | 6 |
| 14 | Piani | 12 | 12 | 66.5 | 0.5 | Italy |  |  | 0 | 32000 | 16000 | 3T | 2x2x2 | 5 |
| 15 | Chuang | 92 | 34 | 15.6 | 0.77 | UK |  |  | 5 | 450 | 750 | 3T | 3x3x3 | 7 |

**g.Healthy**

|  | **First Author** | **Number of participants in the experimental group** | **Number of participants in the control group** | **Age (mean)** | **Male to female ratio** | **Country** | **Total number of trials** | **Proportion of Go trials** | **Stimulus type (others 0, color 1, letter 2, symbol 3, picture 4, word 5)** | **Duration of a single stimulus (ms)** | **Interval between two stimuli (ms)** | **Tesla** | **Voxel**（mm） | **Quality score** |
| --- | --- | --- | --- | --- | --- | --- | --- | --- | --- | --- | --- | --- | --- | --- |
| 1 | Watanabe | 11 | 0 | 25 | 0.8 | Japan | 60 | 0.5 | 1 | 1000 | 11000 | 1.5T | 4x4x8 | 5 |
| 2 | Tamm | 19 | 0 | 14.4 | 0.4 | USA | 156 | 0.5 | 2 | 2000 | 2000 | 1.5T | 0.9x09x1.5 | 5 |
| 3 | Garavan | 16 | 0 | 31 | 0.4 | UK | 1000 | 0.9 | 2 | 750 | 250 | 1.5T | 3.75x3.75x7 | 5 |
| 4 | Mostofsky | 48 | 0 | 27.4 | 0.5 | USA | 300 | 0.8 | 1 | 200 | 1300 | 1.5T | 3.59x3.59x4.5 | 5 |
| 5 | Rubia | 20 | 0 | 28 | 1 | UK | 196 | 0.8 | 3 | 500 | 1800 | 1.5T | 1.5x1.5x3 | 5 |
| 6 | Wood | 16 | 0 | 28.2 | 0.4 | USA | 864 | 0.5 | 1 | 4200 | 3000 | 3T | 0.93x0.93x1.5 | 7 |
| 7 | Maguire | 6 | 0 | 26 | 1 | Netherlands | 240 | 0.5 | 1 | 1000 | 1000 | 1.5T | 3.75x3.75x5.75 | 6 |
| 8 | Horn | 21 | 0 | 34 | 1 | UK | 120 | 0.8 | 2 | 500 | 1395 | 1.5T | 0.89x0.89x3.5 | 3 |
| 9 | Bellgrove | 42 | 0 | 31 | 0.3 | Ireland | 1260 | 0.9 | 2 | 750 | 250 | 1.5T | 3.75x3.75x1 | 4 |
| 10 | Fassbender | 18 | 0 | 26.5 | 0.3 | Ireland | 326 | 0.8 | 0 | 250 | 150 | 1.5T | 3x3x3 | 4 |
| 11 | Kelly | 10 | 0 | 30 | 0 | Ireland | 1252 | 0.9 | 2 | 900 | 100 | 1.5T | 1x1x1 | 4 |
| 12 | Asahi | 17 | 0 | 25.1 | 0.6 | Japan | 288 | 0.5 | 2 | 500 | 1000 | 1.5T | 1x1x1 | 7 |
| 13 | Protopopescu | 12 | 0 | 28 | 0 |  | 480 | 0.6 | 5 | 1000 | 1250 | 3T | 0.94x0.94x1.5 | 5 |
| 14 | Brown | 10 | 0 | 26 | 0.4 | Canada | 1616 | 0.8 | 3 | 500 | 200 | 4T | 0.9x09x1.25 | 4 |
| 15 | Rubia | 52 | 0 | 21.5 | 1 | Britain | 360 | 0.9 | 3 | 500 | 1300 | 1.5T | 3x3x3 | 3 |
| 16 | Passamonti | 24 | 0 | 30.3 | 1 | Italy | 112 | 0.5 | 2 | 300 | 2000 | 1.5T |  | 4 |
| 17 | Pessiglione | 39 | 0 | 28 | 0.6 | Britain | 128 | 0.5 | 5 | 4000 |  | 3T |  | 8 |
| 18 | Chevrier | 14 | 0 | 28.5 | 0.6 | Canada | 322 | 0.8 | 2 | 1000 | 4000 | 1.5T | 3.75x3.75x5 | 4 |
| 19 | Simmonds | 30 | 0 | 10 | 0.4 | USA | 131 | 0.7 | 1 | 300 | 1500 | 1.5T | 3.59x3.59x4.5 | 3 |
| 20 | Rubia | 21 | 26 | 22.3 | 1 | UK | 196 | 0.8 | 3 | 500 | 1800 | 1.5T | 1.5x1.5x3 | 5 |
| 21 | Jimura | 46 | 0 | 23 | 0.6 | Japan | 768 | 0.8 | 3 | 800 |  | 3T | 1x1x2 | 3 |
| 22 | Finger | 20 | 0 | 26 | 0.5 | UK | 384 | 0.5 | 4 | 1100 | 1200 | 3T | 3.75x3.75x3.3 | 7 |
| 23 | Zheng | 20 | 0 | 23 | 0.4 | Japan | 104 | 0.8 | 3 | 1000 | 2000 | 1.5T | 1x1x1 | 5 |
| 24 | Wilbertz | 52 | 0 |  |  | Germany | 480 | 0.7 | 4 | 600 | 5250 | 3T | 1x1x1 | 2 |
| 25 | Chao | 65 | 0 | 35 | 0.5 | USA |  | 0.8 | 4 | 1000 | 3000 | 3T |  | 5 |
| 26 | Chikazoe | 22 | 0 | 22.3 | 0.5 | Japan | 900 | 0.4 | 1 | 800 | 1700 | 1.5T | 2x2x2 | 3 |
| 27 | Chikazoe | 25 | 0 | 23.5 | 0.4 | Japan | 3348 | 0.9 | 1 | 400 | 400 | 1.5T | 2x2x2 | 3 |
| 28 | Bonnet | 20 | 0 | 23.4 | 0.6 | France | 32 | 0.7 | 4 | 1250 | 500 | 1.5T |  | 4 |
| 29 | Callan | 14 | 0 | 27.7 | 0.5 | Japan | 30 |  | 0 | 24000 | 6000 | 1.5T | 1x1x1 | 5 |
| 30 | Li | 33 | 0 | 31 | 0.6 | USA | 420 | 0.8 | 2 | 1000 | 2000 | 3T | 4x4x4 | 5 |
| 31 | Chevrier | 14 | 0 | 29.4 | 0.6 | Canada | 322 | 0.7 | 2 | 1000 | 500 | 1.5T |  | 4 |
| 32 | Simoes | 16 | 0 | 27.5 | 0.3 | UK | 1440 | 0.9 | 3 | 500 | 500 | 1.5T | 4x4x5 | 3 |
| 33 | Hendrick | 60 | 0 | 32 | 0.5 | Japan |  | 0.8 | 4 | 1000 | 2000 | 3T |  | 5 |
| 34 | Jamadar | 24 | 0 | 25 | 0.5 | Australia | 1080 | 0.7 | 2 |  | 700 | 1.5T | 1x1x1 | 4 |
| 35 | Cai | 26 | 0 | 28.5 | 0.6 | USA | 216 | 0.7 | 1 | 400 |  | 3T |  | 5 |
| 36 | Ghahremani | 18 | 0 | 32.5 | 0.6 | USA | 256 | 0.8 | 3 | 500 | 1000 | 1.5T |  | 5 |
| 37 | Spunt | 15 | 0 | 23.5 | 0.4 | USA | 128 | 0.8 | 3 | 1000 | 300 | 3T | 3x3x3 | 3 |
| 38 | Shane | 21 | 0 | 34 | 0.7 | USA | 500 | 0.8 | 2 | 1000 | 1000 | 3T | 3.44x3.44x5 | 5 |
| 39 | Li | 40 | 0 | 32 | 0.5 | USA |  | 0.8 | 2 | 1000 | 2500 | 3T |  | 5 |
| 40 | Chevrier | 14 | 0 | 29.4 |  | Canada | 322 | 0.7 | 2 | 1000 | 1500 | 1.5T | 3.15x3.15x6 | 4 |
| 41 | Xu | 18 | 0 | 26.4 | 0.5 | USA | 400 | 0.8 | 3 | 1500 | 4000 |  |  | 5 |
| 42 | Hu | 114 | 0 | 30.7 | 0.4 | USA | 400 | 0.3 | 3 | 1000 | 2000 | 3T | 4x4x4 | 5 |
| 43 | Hu | 78 | 0 | 30.2 | 0.4 | USA | 400 | 0.3 | 3 | 1000 | 2000 | 3T | 4x4x4 | 5 |
| 44 | Manza | 16 | 0 | 29 | 0.5 | USA |  | 0.5 | 3 | 1000 | 3000 | 3T | 3x3x3 | 5 |

**Table S2：ALE meta-analysis found decreased activities in each disorder: patients vs. healthy controls**

| **SCZ<HC**  **Cluster #** | **x** | **y** | **z** | **P** | **Z** | **Brain region (Brodmann area)** |
| --- | --- | --- | --- | --- | --- | --- |
| 1 | 38 | 20 | 18 | 0 | 3.291 | Right Insula 13 |
| 1 | 36.1 | 17.3 | 11.6 | 0.001 | 3.090 | Right Insula 13 |
| 1 | 30.7 | 20 | -4 | 0.032 | 1.852 | Right Inferior Frontal Gyrus 47 |
| 1 | 35.6 | 27.2 | -11.2 | 0.003 | 2.748 | Right Inferior Frontal Gyrus 47 |
| 1 | 28 | 12 | 6 | 0.004 | 2.652 | Right Claustrum |
| 1 | 35 | 15 | 5 | 0.036 | 1.799 | Right Insula 13 |
| 1 | 49 | 11 | 7 | 0.009 | 2.366 | Right Precentral Gyrus 44 |
| 1 | 44 | 24 | -14 | 0.032 | 1.852 | Right Inferior Frontal Gyrus 47 |
| 2 | -9 | -17 | 2 | 1 | 0.000 | Left Thalamus Mammillary Body |
| 2 | -2 | -24 | 1 | 0.017 | 2.120 | Left Thalamus |
| 2 | 4 | -28 | 4 | 0.02 | 2.054 | Right Thalamus Pulvinar |
| 2 | 8 | -26 | 0 | 0.021 | 2.034 | Right Thalamus |
| 2 | 2 | -20 | 10 | 0.031 | 1.866 | Right Thalamus |
| 2 | 4 | -24 | 12 | 0.034 | 1.825 | Right Thalamus |
| 2 | 4 | -24 | 8 | 0.036 | 1.799 | Right Thalamus Pulvinar |
| 2 | 4 | -18 | 14 | 0.043 | 1.717 | Right Thalamus Medial Dorsal Nucleus |
| 2 | 5.2 | -14 | 13.6 | 0.049 | 1.655 | Right Thalamus Medial Dorsal Nucleus |
| 4 | -48 | 6 | 24 | 0.009 | 2.366 | Left Inferior Frontal Gyrus 9 |
| 4 | -42 | 4 | 22 | 0.004 | 2.652 | Left Inferior Frontal Gyrus 9 |
| 4 | -44 | 6 | 26 | 0.006 | 2.512 | Left Inferior Frontal Gyrus 9 |
| 4 | -40 | 5 | 26 | 0.007 | 2.457 | Left Inferior Frontal Gyrus 9 |
| 4 | -40 | -2 | 28 | 0.008 | 2.409 | Left Precentral Gyrus 6 |
| 4 | -46 | -2 | 32 | 0.011 | 2.290 | Left Precentral Gyrus 6 |
| 4 | -48 | 16 | 32 | 0.031 | 1.866 | Left Middle Frontal Gyrus 9 |
| 5 | -50 | 14 | 0 | 0.006 | 2.512 | Left Inferior Frontal Gyrus |
| 5 | -48 | 7 | 8 | 0.019 | 2.075 | Left Precentral Gyrus 44 |
| 5 | -42 | 20 | -4 | 0.017 | 2.120 | Left Inferior Frontal Gyrus 47 |
| 5 | -46 | 7 | 4 | 0.02 | 2.054 | Left Insula 13 |
| 5 | -40 | 8 | -6 | 0.021 | 2.034 | Left Insula 13 |
| 5 | -37 | 14 | -7 | 0.069 | 0.000 | Left Inferior Frontal Gyrus 47 |
| 5 | -40 | 0 | 6 | 0.033 | 1.838 | Left Insula 13 |
| 5 | -32 | 18 | -8 | 0.035 | 1.812 | Left Inferior Frontal Gyrus 47 |
| 5 | -34 | 14 | -8 | 0.042 | 1.728 | Left Extra-Nuclear 13 |
| 6 | 41 | -61 | 48 | 0.011 | 2.290 | Right Superior Parietal Lobule 7 |
| 6 | 38 | -62 | 52 | 0.004 | 2.652 | Right Superior Parietal Lobule 7 |
| 6 | 44 | -60 | 38 | 0.008 | 2.409 | Right Angular Gyrus 39 |
| 7 | -32 | 52 | 13 | 0.013 | 2.226 | Left Superior Frontal Gyrus 10 |
| 7 | -32 | 52 | 18 | 0.015 | 2.170 | Left Superior Frontal Gyrus 10 |
| 7 | -40 | 46 | 26 | 0.02 | 2.054 | Left Middle Frontal Gyrus 9 |
| 7 | -36 | 44 | 20 | 0.028 | 1.911 | Left Middle Frontal Gyrus 10 |
| 8 | 14 | 10 | 46 | 0.005 | 2.576 | Right Medial Frontal Gyrus 32 |
| 8 | 16 | 8 | 50 | 0.006 | 2.512 | Right Medial Frontal Gyrus 6 |
| 8 | 10 | 10 | 40 | 0.012 | 2.257 | Right Cingulate Gyrus 32 |
| 8 | 4.7 | 5.3 | 35.3 | 0.017 | 2.120 | Right Cingulate Gyrus 24 |
| 9 | 16 | 38 | 36 | 0.015 | 2.170 | Right Superior Frontal Gyrus 9 |
| 9 | 18 | 38 | 40 | 0.016 | 2.144 | Right Superior Frontal Gyrus 8 |
| 10 | 28 | -78 | -10 | 0.01 | 2.326 | Right Lingual Gyrus 18 |
| 10 | 26 | -82 | -10 | 0.014 | 2.197 | Right Fusiform Gyrus 19 |
| 10 | 34.7 | -77.3 | -10 | 0.025 | 1.960 | Right Fusiform Gyrus 19 |
| 10 | 30 | -86 | -6 | 0.038 | 1.774 | Right Inferior Occipital Gyrus 18 |
| 13 | 44 | 39 | 12 | 0.008 | 2.409 | Right Inferior Frontal Gyrus 46 |
| **ADHD<HC**  **Cluster #** | **x** | **y** | **z** | **P** | **Z** | **Brain region (Brodmann area)** |
| 1 | 26.8 | 7 | 24.9 | 1 | 0.000 | No Gray Matter found |
| 1 | 28.4 | 6.3 | 34 | 1 | 0.000 | No Gray Matter found |
| 1 | 27.7 | 7.3 | 28.9 | 1 | 0.000 | No Gray Matter found |
| 1 | 4.6 | 23.5 | 34 | 0.14 | 0.000 | Right Cingulate Gyrus 32 |
| 1 | 42 | 2 | 28 | 0.004 | 2.652 | Right Precentral Gyrus 6 |
| 1 | 44 | 6 | 30 | 0.005 | 2.576 | Right Inferior Frontal Gyrus 9 |
| 1 | -4 | 16 | 38 | 0.006 | 2.512 | Left Cingulate Gyrus 32 |
| 1 | 36 | 22 | 16 | 0.008 | 2.409 | Right Insula 13 |
| 1 | 30 | 14 | 12 | 0.011 | 2.290 | Right Insula 13 |
| 1 | 32 | -10 | 58 | 0.017 | 2.120 | Right Precentral Gyrus 6 |
| 2 | 30.3 | -55.3 | 38 | 0 | 3.291 | Right Angular Gyrus 39 |
| 2 | 25.6 | -65.8 | 35.6 | 0.005 | 2.576 | Right Precuneus 7 |
| 2 | 37.8 | -49.8 | 37.6 | 0.001 | 3.090 | Right Inferior Parietal Lobule 40 |
| 2 | 55.6 | -45.2 | 25.6 | 0.009 | 2.366 | Right Inferior Parietal Lobule 40 |
| 2 | 54.4 | -43.2 | 22.4 | 0.041 | 1.739 | Right Inferior Parietal Lobule 40 |
| 2 | 58.7 | -44 | 22 | 0.018 | 2.097 | Right Inferior Parietal Lobule 40 |
| 2 | 57 | -41 | 29 | 0.009 | 2.366 | Right Inferior Parietal Lobule 40 |
| 2 | 50 | -56 | 33.3 | 0.028 | 1.911 | Right Supramarginal Gyrus 40 |
| 2 | 38 | -60 | 46 | 0.01 | 2.326 | Right Inferior Parietal Lobule 7 |
| 2 | 66 | -28 | 2 | 0.014 | 2.197 | Right Middle Temporal Gyrus 21 |
| 3 | -37.5 | 10.5 | 3 | 0.109 | 0.000 | Left Insula 13 |
| 3 | -28 | 6 | 10 | 0.009 | 2.366 | Left Claustrum |
| 3 | -28 | 11 | 5 | 0.196 | 0.000 | Left Claustrum |
| 3 | -32 | 8 | 14 | 0.017 | 2.120 | Left Insula 13 |
| 3 | -18 | 0 | 6 | 0.015 | 2.170 | Left Putamen |
| 3 | -22 | 0 | 4 | 0.019 | 2.075 | Left Putamen |
| 3 | -19 | 3 | -4 | 1 | 0.000 | Left Putamen |
| 4 | -33.8 | 47.2 | 22.5 | 0 | 3.291 | Left Middle Frontal Gyrus 10 |
| 4 | -32.9 | 47 | 29.8 | 0.001 | 3.090 | Left Superior Frontal Gyrus 9 |
| 5 | 35.3 | 47.3 | 11.3 | 0.002 | 2.878 | Right Middle Frontal Gyrus 10 |
| 5 | 35 | 49.5 | 4.5 | 0.002 | 2.878 | Right Middle Frontal Gyrus 10 |
| 6 | -37.3 | 24 | 35.3 | 0 | 3.291 | Left Precentral Gyrus 9 |
| 6 | -36 | 26 | 41.3 | 0.006 | 2.512 | Left Middle Frontal Gyrus 8 |
| 6 | -42 | 32 | 36 | 0.003 | 2.748 | Left Middle Frontal Gyrus 9 |
| 6 | -24 | 28 | 42 | 0.005 | 2.576 | Left Middle Frontal Gyrus 8 |
| 7 | -48 | -8 | 46 | 0 | 3.291 | Left Precentral Gyrus 4 |
| 7 | -46 | -8 | 42 | 0.002 | 2.878 | Left Precentral Gyrus 4 |
| 7 | -46 | -4 | 44 | 0.003 | 2.748 | Left Precentral Gyrus 6 |
| 7 | -44 | -4 | 40 | 0.005 | 2.576 | Left Precentral Gyrus 6 |
| 7 | -42 | -12 | 48 | 0.01 | 2.326 | Left Precentral Gyrus 4 |
| 7 | -42 | 0 | 22 | 0.014 | 2.197 | Left Inferior Frontal Gyrus 9 |
| 7 | -40 | -4 | 28 | 0.016 | 2.144 | Left Precentral Gyrus 6 |
| 7 | -42 | 8 | 24 | 0.021 | 2.034 | Left Inferior Frontal Gyrus 9 |
| 7 | -44 | -4 | 30 | 0.025 | 1.960 | Left Precentral Gyrus 6 |
| 7 | -36 | 0 | 32 | 0.031 | 1.866 | Left Precentral Gyrus 6 |
| 9 | 51.3 | -48.2 | -1.3 | 0 | 3.291 | Right Middle Temporal Gyrus 22 |
| 10 | -32 | -84 | -6 | 0.005 | 2.576 | Left Inferior Occipital Gyrus 18 |
| 10 | -34 | -88 | -7 | 0.006 | 2.512 | Left Inferior Occipital Gyrus 18 |
| 10 | -30 | -90 | -12 | 0.009 | 2.366 | Left Inferior Occipital Gyrus 18 |
| 10 | -30 | -88 | -16 | 0.013 | 2.226 | Left Fusiform Gyrus 18 |
| 10 | -24 | -92 | -10 | 0.015 | 2.170 | Left Fusiform Gyrus 18 |
| 11 | -27.3 | -10 | 50.7 | 0.005 | 2.576 | Left Precentral Gyrus 6 |
| 11 | -30.7 | -8 | 56 | 0.006 | 2.512 | Left Precentral Gyrus 6 |
| 11 | -22 | -10 | 46 | 0.009 | 2.366 | Left Middle Frontal Gyrus 6 |
| 12 | 4 | -36 | 30 | 0.006 | 2.512 | Right Cingulate Gyrus 31 |
| 12 | 4 | -36 | 22 | 0.007 | 2.457 | Right Posterior Cingulate 23 |
| 13 | -39.9 | -52.5 | 47.6 | 0 | 3.291 | Left Inferior Parietal Lobule 40 |
| 14 | -48 | 37 | 8 | 0.008 | 2.409 | Left Inferior Frontal Gyrus 46 |
| 14 | -48 | 40 | 14 | 0.016 | 2.144 | Left Inferior Frontal Gyrus 46 |
| 14 | -46 | 38 | 4 | 0.018 | 2.097 | Left Inferior Frontal Gyrus 45 |
| 14 | -46 | 42 | 6 | 0.02 | 2.054 | Left Inferior Frontal Gyrus 46 |
| 15 | -4 | 46 | 34 | 0.008 | 2.409 | Left Medial Frontal Gyrus 6 |
| 15 | -14 | 48 | 38 | 0.022 | 2.014 | Left Superior Frontal Gyrus 8 |
| 15 | -10 | 44 | 42 | 0.035 | 1.812 | Left Superior Frontal Gyrus 8 |
| 16 | 42 | -24 | -6 | 0.017 | 2.120 | Right Superior Temporal Gyrus 22 |
| 16 | 44 | -22 | -10 | 0.03 | 1.881 | Right Superior Temporal Gyrus 22 |
| 16 | 46 | -32 | -4 | 0.045 | 1.695 | Right Middle Temporal Gyrus 21 |
| 18 | -56 | -34 | 16 | 0.004 | 2.652 | Left Insula 13 |
| 19 | -56 | -48 | 4 | 0.002 | 2.878 | Left Middle Temporal Gyrus 22 |
| 20 | -22 | -69 | 41 | 0.028 | 1.911 | Left Precuneus 7 |
| 20 | -20 | -68 | 32 | 0.029 | 1.896 | Left Precuneus 7 |
| 21 | -21 | -4 | -16 | 0.032 | 1.852 | Left Amygdala |
| 21 | -16 | -8 | -12 | 0.035 | 1.812 | Left Amygdala |
| 21 | -18 | -4 | -12 | 0.039 | 1.762 | Left Amygdala |
| 22 | 4 | -28 | -4 | 0.014 | 2.197 | Right Thalamus |
| 23 | 38 | -78 | -8 | 0.017 | 2.120 | Right Middle Occipital Gyrus 18 |
| 23 | 34.5 | -81 | -7.5 | 0.034 | 1.825 | Right Middle Occipital Gyrus 18 |
| **ASD<HC**  **Cluster #** | **x** | **y** | **z** | **P** | **Z** | **Brain region (Brodmann area)** |
| 1 | 18.6 | -1.7 | -0.7 | 1 | 0.000 | Right Lateral Globus Pallidus |
| 1 | 11.2 | -10.3 | 8.3 | 0.02 | 2.054 | Right ThalamusVentral Lateral Nucleus |
| 1 | 24.2 | 2.9 | -0.5 | 0.166 | 0.000 | Right Putamen |
| 1 | -8 | -23 | 0 | 0.003 | 2.748 | Left Thalamus |
| 1 | 14 | 13 | 6 | 0.017 | 2.120 | Right CaudateCaudate Body |
| 1 | 20 | -2 | 0 | 0.013 | 2.226 | Right Lateral Globus Pallidus |
| 1 | 20 | 2 | -6 | 0.014 | 2.197 | Right Putamen |
| 1 | 10 | 14 | 10 | 0.023 | 1.995 | Right Caudate Caudate Body |
| 2 | 52.9 | -41.3 | 28 | 0 | 3.291 | Right Inferior Parietal Lobule 40 |
| 2 | 50.8 | -36.1 | 12.9 | 1 | 0.000 | Right Superior Temporal Gyrus 41 |
| 2 | 50.8 | -26.8 | 5 | 0.002 | 2.878 | Right Superior Temporal Gyrus 41 |
| 2 | 52.2 | -18.2 | -0.5 | 0.003 | 2.748 | Right Superior Temporal Gyrus 22 |
| 2 | 42 | -49 | 48 | 1 | 0.000 | Right Inferior Parietal Lobule 40 |
| 2 | 54 | -38 | 8 | 0.015 | 2.170 | Right Superior Temporal Gyrus 22 |
| 2 | 36 | -54 | 52 | 0.031 | 1.866 | Right Superior Parietal Lobule 7 |
| 3 | -34.1 | 29.1 | 36.3 | 0.005 | 2.576 | Left Middle Frontal Gyrus 9 |
| 3 | -31.4 | 45.2 | 20.6 | 0.003 | 2.748 | Left Middle Frontal Gyrus 10 |
| 3 | -31.8 | 44.4 | 27.7 | 0.011 | 2.290 | Left Superior Frontal Gyrus 9 |
| 3 | -36.8 | 46.2 | 23.2 | 0.003 | 2.748 | Left Middle Frontal Gyrus 10 |
| 3 | -40 | 40 | 10 | 0.004 | 2.652 | Left Middle Frontal Gyrus 10 |
| 3 | -46 | 10 | 22 | 0.007 | 2.457 | Left Inferior Frontal Gyrus 9 |
| 4 | 28.2 | 49 | 13.8 | 0 | 3.291 | Right Superior Frontal Gyrus 10 |
| 4 | 16.4 | 45 | 33.9 | 0.003 | 2.748 | Right Superior Frontal Gyrus 9 |
| 4 | 25.6 | 49.2 | 12.4 | 0 | 3.291 | Right Superior Frontal Gyrus 10 |
| 5 | 41.3 | -6.3 | 45.9 | 0 | 3.291 | Right Precentral Gyrus 6 |
| 5 | 35.4 | -5.6 | 44.8 | 0.001 | 3.090 | Right Middle Frontal Gyrus 6 |
| 5 | 44.4 | 2.8 | 46.8 | 0.002 | 2.878 | Right Middle Frontal Gyrus 6 |
| 5 | 32 | -2 | 34 | 0.003 | 2.748 | Right Precentral Gyrus 6 |
| 5 | 36 | 12 | 34 | 0.008 | 2.409 | Right Precentral Gyrus 9 |
| 5 | 23 | -12 | 54 | 0.046 | 1.685 | Right Middle Frontal Gyrus 6 |
| 6 | -1.5 | -4.5 | 48.4 | 0 | 3.291 | Left Medial Frontal Gyrus 6 |
| 6 | 0 | -7 | 53 | 0.001 | 3.090 | Left Medial Frontal Gyrus 6 |
| 6 | -28 | -4 | 50 | 0.004 | 2.652 | Left Middle Frontal Gyrus 6 |
| 6 | -42 | -12 | 48 | 0.005 | 2.576 | Left Precentral Gyrus 4 |
| 6 | -16 | -12 | 54 | 0.006 | 2.512 | Left Medial Frontal Gyrus 6 |
| 6 | -38 | -8 | 48 | 0.007 | 2.457 | Left Precentral Gyrus 6 |
| 6 | -42 | -6 | 48 | 0.009 | 2.366 | Left Precentral Gyrus 6 |
| 6 | -27 | -8 | 49 | 0.024 | 1.977 | Left Precentral Gyrus 6 |
| 6 | -32 | -8 | 56 | 0.026 | 1.943 | Left Precentral Gyrus 6 |
| 6 | -8 | -14 | 64 | 0.033 | 1.838 | Left Superior Frontal Gyrus 6 |
| 7 | -45.7 | -65.1 | 0.3 | 0.001 | 3.090 | Left Inferior Temporal Gyrus 37 |
| 7 | -39.7 | -72.8 | -1.4 | 0.001 | 3.090 | Left Inferior Occipital Gyrus 19 |
| 7 | -47.8 | -66 | -5.8 | 0.002 | 2.878 | Left Middle Occipital Gyrus 37 |
| 8 | -54.7 | -44.1 | 20.7 | 0 | 3.291 | Left Superior Temporal Gyrus 13 |
| 8 | -56.5 | -41.1 | 12.6 | 1 | 0.000 | Left Superior Temporal Gyrus 22 |
| 9 | 9.3 | -70.9 | 36.8 | 0 | 3.291 | Right Precuneus 7 |
| 9 | 12.4 | -74.1 | 41.6 | 0.001 | 3.090 | Right Precuneus 7 |
| 9 | 17.6 | -72.1 | 36.1 | 0.002 | 2.878 | Right Precuneus 7 |
| 9 | 18.5 | -72.5 | 44 | 0.003 | 2.748 | Right Precuneus 7 |
| 9 | 26 | -72 | 28 | 0.023 | 1.995 | Right Precuneus 31 |
| 10 | -43.4 | 16.4 | 2.7 | 0 | 3.291 | Left Insula 13 |
| 10 | -50 | 6 | 8 | 0.001 | 3.090 | Left Precentral Gyrus 44 |
| 10 | -48 | 2 | 6 | 0.002 | 2.878 | Left Precentral Gyrus 44 |
| 10 | -38 | 8 | 0 | 0.003 | 2.748 | Left Insula 13 |
| 11 | -41.2 | -45.1 | 38.7 | 0 | 3.291 | Left Inferior Parietal Lobule 40 |
| 12 | 4 | -34 | 24 | 0.002 | 2.878 | Right Posterior Cingulate 23 |
| 12 | -4 | -34 | 26 | 0.005 | 2.576 | Left Cingulate Gyrus 23 |
| 12 | 4 | -40 | 28 | 0.008 | 2.409 | Right Cingulate Gyrus 31 |
| 13 | -12 | -64 | 50 | 0 | 3.291 | Left Precuneus 7 |
| 13 | -11 | -65 | 45 | 0.001 | 3.090 | Left Precuneus 7 |
| 13 | -11 | -70 | 29.5 | 0.002 | 2.878 | Left Precuneus 31 |
| 13 | -16 | -66 | 48 | 0.003 | 2.748 | Left Precuneus 7 |
| 13 | -12 | -69.5 | 36 | 1 | 0.000 | Left Precuneus 7 |
| 13 | -22 | -64 | 42 | 0.008 | 2.409 | Left Superior Parietal Lobule 7 |
| 14 | -55 | -30 | 3 | 0.002 | 2.878 | Left Superior Temporal Gyrus 22 |
| 14 | -51.8 | -26.4 | 1.2 | 0.002 | 2.878 | Left Superior Temporal Gyrus 22 |
| 14 | -56 | -30 | -4 | 0.003 | 2.748 | Left Middle Temporal Gyrus 21 |
| 14 | -53 | -25 | -4 | 0.019 | 2.075 | Left Middle Temporal Gyrus 21 |
| 15 | 46 | 4 | -2 | 0.013 | 2.226 | Right Insula 13 |
| 15 | 51 | 10 | 5 | 1 | 0.000 | Right Precentral Gyrus 44 |
| 16 | 15.5 | -31.7 | -2.2 | 0 | 3.291 | Right Thalamus |
| 16 | 22 | -28 | -4 | 0.001 | 3.090 | Right 27 |
| 17 | -9.5 | 46 | 38.5 | 0.002 | 2.878 | Left Superior Frontal Gyrus 8 |
| 17 | -5 | 46.1 | 36.6 | 0.001 | 3.090 | Left Medial Frontal Gyrus 6 |
| 17 | -10 | 50 | 38 | 0.002 | 2.878 | Left Superior Frontal Gyrus 8 |
| 18 | 60.4 | -28.8 | 23.2 | 0.007 | 2.457 | Right Inferior Parietal Lobule 40 |
| 19 | -42 | -25.6 | 11.6 | 0 | 3.291 | Left Transverse Temporal Gyrus 41 |
| 19 | -44 | -22 | 16 | 0.001 | 3.090 | Left Insula 13 |
| 20 | -52 | -26 | 36 | 0 | 3.291 | Left Postcentral Gyrus 2 |
| 20 | -52 | -26 | 40 | 0.001 | 3.090 | Left Postcentral Gyrus 2 |
| 20 | -52 | -20 | 38 | 0.004 | 2.652 | Left Postcentral Gyrus 3 |
| 20 | -52 | -16 | 40 | 0.006 | 2.512 | Left Postcentral Gyrus 3 |
| 21 | -22 | -3 | 5 | 0.037 | 1.787 | Left Putamen |
| 21 | -22 | -3 | 6 | 0.037 | 1.787 | Left Putamen |
| 21 | -24 | 0 | 0 | 0.038 | 1.774 | Left Putamen |
| 22 | 53.2 | -7.6 | -10.8 | 0.005 | 2.576 | Right Superior Temporal Gyrus 21 |
| 23 | -8.3 | 38.9 | 46.5 | 0.001 | 3.090 | Left Superior Frontal Gyrus 8 |
| **BD<HC**  **Cluster #** | **x** | **y** | **z** | **P** | **Z** | **Brain region (Brodmann area)** |
| 1 | 27.1 | 7.7 | 28.8 | 1 | 0.000 | No Gray Matter found |
| 1 | 31.3 | 7.3 | 24.2 | 1 | 0.000 | Right Precentral Gyrus 6 |
| 1 | 10 | 14.7 | 14 | 1 | 0.000 | Right CaudateCaudate Body |
| 1 | 38 | 14 | 16 | 0.005 | 2.576 | Right Insula 13 |
| 1 | -10 | -10 | 64 | 0.007 | 2.457 | Left Medial Frontal Gyrus 6 |
| 1 | 21 | -3 | 48 | 0.016 | 2.144 | Right Middle Frontal Gyrus 6 |
| 1 | 28 | -6 | 42 | 0.012 | 2.257 | Right Middle Frontal Gyrus 6 |
| 1 | 16 | -2 | 64 | 0.019 | 2.075 | Right Superior Frontal Gyrus 6 |
| 1 | 24 | -13.5 | 58 | 0.031 | 1.866 | Right Middle Frontal Gyrus 6 |
| 1 | -20 | -11 | 49 | 0.034 | 1.825 | Left Middle Frontal Gyrus 6 |
| 2 | 31.3 | 33.5 | 35 | 0.001 | 3.090 | Right Middle Frontal Gyrus 9 |
| 2 | 35.9 | 26.2 | 34.1 | 0.001 | 3.090 | Right Middle Frontal Gyrus 9 |
| 2 | 34 | 26 | 24 | 0.002 | 2.878 | No Gray Matter found |
| 3 | 14.5 | -13.5 | 9.5 | 0.001 | 3.090 | Right ThalamusVentral Lateral Nucleus |
| 3 | 6 | -15 | 16 | 0.003 | 2.748 | Right Thalamus |
| 3 | 10 | -25 | 0 | 0.004 | 2.652 | Right Thalamus |
| 3 | 0 | -28 | -12 | 0.006 | 2.512 | No Gray Matter found |
| 4 | -46 | 2 | 20 | 0.001 | 3.090 | Left Inferior Frontal Gyrus 9 |
| 4 | -44 | -2 | 24 | 0.002 | 2.878 | Left Inferior Frontal Gyrus 9 |
| 4 | -42.5 | 2.4 | 30.7 | 0.003 | 2.748 | Left Inferior Frontal Gyrus 9 |
| 4 | -45 | 8 | 36 | 1 | 0.000 | Left Middle Frontal Gyrus 9 |
| 4 | -42.7 | 3.3 | 36.7 | 1 | 0.000 | Left Precentral Gyrus 6 |
| 4 | -44 | 25 | 33 | 0.007 | 2.457 | Left Middle Frontal Gyrus 9 |
| 4 | -38 | 20 | 34 | 0.01 | 2.326 | Left Middle Frontal Gyrus 9 |
| 4 | -40 | 12 | 32 | 0.017 | 2.120 | Left Middle Frontal Gyrus 9 |
| 4 | -36 | 26 | 42 | 0.023 | 1.995 | Left Middle Frontal Gyrus 8 |
| 5 | -38.7 | -59.5 | -11.8 | 0 | 3.291 | Left Fusiform Gyrus 37 |
| 5 | -48 | -70 | 0 | 0.007 | 2.457 | Left Inferior Temporal Gyrus |
| 6 | 54 | -46 | 40 | 0 | 3.291 | Right Inferior Parietal Lobule 40 |
| 6 | 52 | -43 | 44 | 0.001 | 3.090 | Right Inferior Parietal Lobule 40 |
| 6 | 51 | -47 | 35 | 0.004 | 2.652 | Right Supramarginal Gyrus 40 |
| 6 | 49 | -45 | 37 | 0.003 | 2.748 | Right Inferior Parietal Lobule 40 |
| 6 | 48 | -58 | 32 | 0.009 | 2.366 | Right Superior Temporal Gyrus 39 |
| 6 | 58 | -38 | 24 | 0.032 | 1.852 | Right Inferior Parietal Lobule 40 |
| 7 | 28.7 | -56.5 | 34.9 | 0 | 3.291 | Right Sub-Gyral 39 |
| 7 | 34 | -65 | 46 | 0.004 | 2.652 | Right Superior Parietal Lobule 7 |
| 8 | -44.3 | 3.7 | 5.5 | 0.001 | 3.090 | Left Insula 13 |
| 8 | -50 | 8 | 1 | 0.003 | 2.748 | Left Superior Temporal Gyrus 22 |
| 8 | -32 | 14 | -8 | 0.015 | 2.170 | Left Extra-Nuclear 13 |
| 8 | -36 | 8 | -8 | 0.022 | 2.014 | Left Extra-Nuclear 13 |
| 9 | -60 | -45.1 | 14 | 0.002 | 2.878 | Left Superior Temporal Gyrus 22 |
| 9 | -54 | -46 | 10.8 | 0.005 | 2.576 | Left Superior Temporal Gyrus 22 |
| 9 | -56 | -28 | 16 | 0.005 | 2.576 | Left Superior Temporal Gyrus 42 |
| 9 | -54 | -38 | 16 | 0.018 | 2.097 | Left Insula 13 |
| 9 | -62 | -52 | 28 | 0.024 | 1.977 | Left Supramarginal Gyrus 40 |
| 9 | -56 | -54 | 30 | 0.03 | 1.881 | Left Supramarginal Gyrus 40 |
| 9 | -60 | -52 | 32 | 0.031 | 1.866 | Left Supramarginal Gyrus 40 |
| 10 | -24 | 48 | 36 | 0.004 | 2.652 | Left Superior Frontal Gyrus 9 |
| 10 | -23 | 49 | 32 | 1 | 0.000 | Left Superior Frontal Gyrus 9 |
| 10 | -28 | 46 | 38 | 0.007 | 2.457 | Left Superior Frontal Gyrus 8 |
| 10 | -32 | 44 | 36 | 0.01 | 2.326 | Left Middle Frontal Gyrus 9 |
| 10 | -32 | 47.7 | 30.7 | 0.013 | 2.226 | Left Superior Frontal Gyrus 9 |
| 11 | 40 | -44 | -16 | 0.006 | 2.512 | Right Fusiform Gyrus 37 |
| 11 | 44.7 | -47.3 | -8.7 | 1 | 0.000 | Right Sub-Gyral 37 |
| 11 | 36 | -46 | -17 | 0.009 | 2.366 | Right Culmen |
| 11 | 48 | -54 | -8 | 0.01 | 2.326 | Right Fusiform Gyrus 37 |
| 11 | 48 | -58 | -8 | 0.011 | 2.290 | Right Middle Occipital Gyrus 19 |
| 11 | 52 | -48 | -2 | 0.012 | 2.257 | Right Sub-Gyral 37 |
| 11 | 44 | -48 | -14 | 0.016 | 2.144 | Right Fusiform Gyrus 37 |
| 11 | 44 | -46 | -18 | 0.014 | 2.197 | Right Culmen |
| 11 | 44 | -66 | -8 | 0.029 | 1.896 | Right Fusiform Gyrus 19 |
| 12 | 0 | 34 | 30 | 0.004 | 2.652 | Left Medial Frontal Gyrus 9 |
| 12 | 4 | 30 | 32 | 0.009 | 2.366 | Right Cingulate Gyrus 32 |
| 12 | 10 | 34 | 30 | 0.019 | 2.075 | Right Cingulate Gyrus 32 |
| 13 | 4 | -32 | 34 | 0.009 | 2.366 | Right Cingulate Gyrus 31 |
| 13 | 0 | -34 | 32 | 0.015 | 2.170 | Left Cingulate Gyrus 31 |
| 13 | 0 | -28 | 24 | 0.027 | 1.927 | Left Cingulate Gyrus 23 |
| 13 | -1.3 | -24.7 | 26.7 | 0.035 | 1.812 | Left Cingulate Gyrus 23 |
| 14 | 62 | -30 | 8 | 0.004 | 2.652 | Right Superior Temporal Gyrus 42 |
| 14 | 61 | -34 | 7 | 1 | 0.000 | Right Superior Temporal Gyrus 22 |
| 14 | 62 | -26 | 8 | 0.006 | 2.512 | Right Superior Temporal Gyrus 42 |
| 14 | 60 | -42 | 5.3 | 0.013 | 2.226 | Right Middle Temporal Gyrus 22 |
| 14 | 56 | -46 | 6 | 0.024 | 1.977 | Right Middle Temporal Gyrus 21 |
| 14 | 58 | -40 | 16 | 0.036 | 1.799 | Right Superior Temporal Gyrus 13 |
| 15 | 37 | 51 | 1 | 0.014 | 2.197 | Right Middle Frontal Gyrus 10 |
| 15 | 31.8 | 50.6 | -0.8 | 0.015 | 2.170 | Right Middle Frontal Gyrus 10 |
| 16 | 46 | -16 | 2 | 0.003 | 2.748 | Right Superior Temporal Gyrus 22 |
| 16 | 58 | -14 | 2 | 0.01 | 2.326 | Right Superior Temporal Gyrus |
| 16 | 56 | -18 | 4 | 0.017 | 2.120 | Right Superior Temporal Gyrus 41 |
| 17 | -41 | -46 | 44 | 0.002 | 2.878 | Left Inferior Parietal Lobule 40 |
| 17 | -42 | -48 | 50 | 0.002 | 2.878 | Left Inferior Parietal Lobule 40 |
| 17 | -42 | -54 | 42 | 0.004 | 2.652 | Left Inferior Parietal Lobule 40 |
| 17 | -44 | -36 | 38 | 0.024 | 1.977 | Left Inferior Parietal Lobule 40 |
| 19 | -58 | -28 | 2 | 0.013 | 2.226 | Left Middle Temporal Gyrus 21 |
| 19 | -58 | -32 | 0 | 0.014 | 2.197 | Left Middle Temporal Gyrus |
| 19 | -56 | -24 | 2 | 0.016 | 2.144 | Left Superior Temporal Gyrus 22 |
| 19 | -56 | -22 | -2 | 0.03 | 1.881 | Left Middle Temporal Gyrus 21 |
| 20 | 37 | -78 | -8 | 0.008 | 2.409 | Right Middle Occipital Gyrus 18 |
| 22 | -38 | -30 | 46 | 0 | 3.291 | Left Postcentral Gyrus 40 |
| 22 | -37.5 | -25.5 | 42.5 | 0.001 | 3.090 | Left Postcentral Gyrus 2 |
| **MDD<HC**  **Cluster #** | **x** | **y** | **z** | **P** | **Z** | **Brain region (Brodmann area)** |
| 1 | 37.9 | 8.5 | 24 | 0 | 3.291 | Right Inferior Frontal Gyrus 9 |
| 1 | 8.3 | -1.9 | 41.8 | 0.001 | 3.090 | Right Cingulate Gyrus 24 |
| 1 | 21.1 | 5.1 | 34.9 | 1 | 0.000 | Right Cingulate Gyrus 24 |
| 1 | 7.4 | 7.7 | 36.4 | 0.003 | 2.748 | Right Cingulate Gyrus 24 |
| 1 | 18.7 | 3.8 | 45.3 | 1 | 0.000 | Right Medial Frontal Gyrus 6 |
| 1 | 9.7 | 3.4 | 51.6 | 0.005 | 2.576 | Right Medial Frontal Gyrus 6 |
| 1 | 13.3 | 8.7 | 47.7 | 0.005 | 2.576 | Right Cingulate Gyrus 24 |
| 1 | 10.5 | 9 | 43 | 0.005 | 2.576 | Right Medial Frontal Gyrus 32 |
| 1 | -8 | -16 | 52 | 0.008 | 2.409 | Left Medial Frontal Gyrus 6 |
| 1 | 37 | 4 | 26 | 0.007 | 2.457 | Right Precentral Gyrus 6 |
| 1 | 16 | -6 | 64 | 0.013 | 2.226 | Right Superior Frontal Gyrus 6 |
| 1 | 14 | -12 | 62 | 0.014 | 2.197 | Right Superior Frontal Gyrus 6 |
| 1 | 18 | -14 | 64 | 0.033 | 1.838 | Right Superior Frontal Gyrus 6 |
| 1 | 28 | 8 | 52 | 0.037 | 1.787 | Right Middle Frontal Gyrus 6 |
| 1 | 30 | 14 | 38 | 0.041 | 1.739 | Right Middle Frontal Gyrus 8 |
| 2 | 27 | -68.5 | 36 | 0.001 | 3.090 | Right Precuneus 7 |
| 2 | 24.6 | -64 | 32.9 | 0.002 | 2.878 | Right Precuneus 7 |
| 2 | 25.4 | -67.8 | 40.2 | 0.002 | 2.878 | Right Precuneus 7 |
| 2 | 22 | -72 | 38 | 0.003 | 2.748 | Right Precuneus 7 |
| 2 | 22.8 | -65.2 | 40.8 | 0.004 | 2.652 | Right Precuneus 7 |
| 2 | 12 | -68 | 42 | 0.006 | 2.512 | Right Precuneus 7 |
| 2 | 10 | -73 | 35 | 0.01 | 2.326 | Right Precuneus 7 |
| 2 | 26 | -64 | 32 | 0.001 | 3.090 | Right Precuneus 7 |
| 2 | 8 | -70 | 35 | 0.009 | 2.366 | Right Precuneus 7 |
| 2 | 8 | -68 | 46 | 0.01 | 2.326 | Right Precuneus 7 |
| 2 | 44 | -58 | 34 | 0.011 | 2.290 | Right Angular Gyrus 39 |
| 2 | 46 | -52 | 34 | 0.013 | 2.226 | Right Inferior Parietal Lobule 40 |
| 2 | 38 | -66 | 50 | 0.015 | 2.170 | Right Superior Parietal Lobule 7 |
| 2 | 54 | -44 | 40 | 0.03 | 1.881 | Right Inferior Parietal Lobule 40 |
| 3 | -47.6 | 28.7 | 10.6 | 1 | 0.000 | Left Inferior Frontal Gyrus 46 |
| 3 | -46.8 | 33.2 | 14 | 0.006 | 2.512 | Left Middle Frontal Gyrus 46 |
| 3 | -40 | 37.1 | 16.7 | 1 | 0.000 | Left Middle Frontal Gyrus 46 |
| 3 | -39.5 | 39.5 | 15.8 | 1 | 0.000 | Left Middle Frontal Gyrus 10 |
| 3 | -37.5 | 36.3 | 29.8 | 1 | 0.000 | Left Superior Frontal Gyrus 9 |
| 3 | -32.5 | 35.5 | 31.5 | 0.006 | 2.512 | Left Superior Frontal Gyrus 9 |
| 3 | -29 | 40 | 36 | 0.01 | 2.326 | Left Middle Frontal Gyrus 9 |
| 3 | -32.4 | 32.7 | 33.1 | 0.009 | 2.366 | Left Middle Frontal Gyrus 9 |
| 3 | -36 | 39 | 32 | 0.007 | 2.457 | Left Superior Frontal Gyrus 9 |
| 3 | -32 | 36 | 38 | 0.009 | 2.366 | Left Middle Frontal Gyrus 9 |
| 3 | -30 | 40.7 | 34.3 | 0.013 | 2.226 | Left Middle Frontal Gyrus 9 |
| 3 | -40 | 48 | 11 | 0.012 | 2.257 | Left Middle Frontal Gyrus 10 |
| 3 | -26 | 50 | 32 | 0.013 | 2.226 | Left Superior Frontal Gyrus 9 |
| 3 | -26 | 47 | 37 | 1 | 0.000 | Left Superior Frontal Gyrus 9 |
| 3 | -34 | 50 | 20 | 0.016 | 2.144 | Left Superior Frontal Gyrus 10 |
| 3 | -44 | 48 | 6 | 0.018 | 2.097 | Left Middle Frontal Gyrus 10 |
| 3 | -46 | 48 | 2 | 0.038 | 1.774 | Left Inferior Frontal Gyrus 46 |
| 4 | -26 | 1.6 | 4.1 | 0 | 3.291 | Left Putamen |
| 4 | -35.6 | 13.6 | 8.8 | 0.003 | 2.748 | Left Insula 13 |
| 4 | -26 | 0 | -1.3 | 0 | 3.291 | Left Putamen |
| 4 | -25 | 2 | -3 | 0 | 3.291 | Left Putamen |
| 4 | -16 | 0 | 18 | 0.015 | 2.170 | Left Caudate Body |
| 5 | 2 | -16 | 34 | 0.003 | 2.748 | Right Cingulate Gyrus 24 |
| 5 | 4 | -38 | 28 | 0.004 | 2.652 | Right Cingulate Gyrus 31 |
| 5 | 4 | -20 | 34 | 0.005 | 2.576 | Right Cingulate Gyrus 24 |
| 5 | 6 | -16 | 30 | 0.006 | 2.512 | Right Cingulate Gyrus 23 |
| 5 | 8 | -40 | 26 | 0.01 | 2.326 | Right Posterior Cingulate 23 |
| 5 | 2 | -40 | 24 | 0.013 | 2.226 | Right Posterior Cingulate 23 |
| 5 | 4 | -24 | 34 | 0.014 | 2.197 | Right Cingulate Gyrus 24 |
| 5 | -2 | -22 | 32 | 0.015 | 2.170 | Left Cingulate Gyrus 23 |
| 5 | 0 | -22 | 28 | 0.019 | 2.075 | Left Cingulate Gyrus 23 |
| 5 | 4 | -26 | 26 | 0.021 | 2.034 | Right Cingulate Gyrus 23 |
| 5 | 4 | -26 | 30 | 0.023 | 1.995 | Right Cingulate Gyrus 23 |
| 5 | -2 | -28 | 26 | 0.031 | 1.866 | Left Cingulate Gyrus 23 |
| 5 | 4 | -30 | 28 | 0.037 | 1.787 | Right Cingulate Gyrus 23 |
| 5 | -4 | -34 | 30 | 0.04 | 1.751 | Left Cingulate Gyrus 31 |
| 5 | 2 | -30 | 32 | 0.042 | 1.728 | Right Cingulate Gyrus 31 |
| 6 | 21 | 36 | 34 | 0.018 | 2.097 | Right Superior Frontal Gyrus 9 |
| 6 | 19.5 | 41 | 37 | 0.02 | 2.054 | Right Superior Frontal Gyrus 9 |
| 7 | 12 | -18 | 18 | 0.021 | 2.034 | Right ThalamusLateral Dorsal Nucleus |
| 7 | 18 | -10 | 8 | 0.03 | 1.881 | Right ThalamusVentral Lateral Nucleus |
| 7 | 18 | -12 | 12 | 0.031 | 1.866 | Right ThalamusVentral Lateral Nucleus |
| 8 | 44 | -58 | -8 | 0.027 | 1.927 | Right Fusiform Gyrus 37 |
| 9 | -38 | -58 | -6 | 0.041 | 1.739 | Left Fusiform Gyrus 37 |
| 10 | 44 | -52 | -12 | 0.027 | 1.927 | Right Fusiform Gyrus 37 |
| 10 | 44 | -46 | -14 | 0.028 | 1.911 | Right Fusiform Gyrus 37 |
| 10 | 44 | -47 | -14 | 0.028 | 1.911 | Right Fusiform Gyrus 37 |
| 11 | -10 | -70 | 30 | 0.015 | 2.170 | Left Cuneus 7 |
| 11 | -10 | -72 | 34 | 0.017 | 2.120 | Left Precuneus 7 |
| **OCD<HC**  **Cluster #** | **x** | **y** | **z** | **P** | **Z** | **Brain region (Brodmann area)** |
| 1 | 42 | -56 | 24 | 0.007 | 2.457 | Right Middle Temporal Gyrus 39 |
| 1 | 30 | -66 | 34 | 0.008 | 2.409 | Right Precuneus 7 |
| 1 | 36 | -57 | 38 | 0.009 | 2.366 | Right Angular Gyrus 39 |
| 1 | 39.3 | -56.7 | 33.3 | 0.011 | 2.290 | Right Angular Gyrus 39 |
| 1 | 39.6 | -61.6 | 42.4 | 0.011 | 2.290 | Right Inferior Parietal Lobule 7 |
| 1 | 32 | -57.2 | 31.6 | 0.013 | 2.226 | Right Middle Temporal Gyrus 39 |
| 1 | 46.7 | -58.7 | 34.7 | 0.014 | 2.197 | Right Angular Gyrus 39 |
| 1 | 39 | -64 | 34 | 1 | 0.000 | Right Precuneus 39 |
| 1 | 46 | -58 | 43.3 | 0.016 | 2.144 | Right Inferior Parietal Lobule 40 |
| 1 | 26 | -68 | 28 | 0.02 | 2.054 | Right Precuneus 7 |
| 1 | 35 | -63.5 | 50.5 | 0.022 | 2.014 | Right Superior Parietal Lobule 7 |
| 1 | 52 | -58 | 30 | 0.023 | 1.995 | Right Superior Temporal Gyrus 39 |
| 1 | 26 | -74 | 40 | 0.029 | 1.896 | Right Precuneus 7 |
| 1 | 42 | -58 | 38 | 0.013 | 2.226 | Right Angular Gyrus 39 |
| 1 | 20 | -70 | 46 | 0.039 | 1.762 | Right Precuneus 7 |
| 1 | 18 | -71 | 41 | 0.043 | 1.717 | Right Precuneus 7 |
| 1 | 20 | -74 | 44 | 0.044 | 1.706 | Right Precuneus 7 |
| 2 | 40 | 6 | 22 | 0.016 | 2.144 | Right Inferior Frontal Gyrus 9 |
| 2 | 40 | 10 | 22 | 0.019 | 2.075 | Right Inferior Frontal Gyrus 9 |
| 2 | 36 | -6 | 32 | 0.02 | 2.054 | Right Precentral Gyrus 6 |
| 2 | 46 | 7 | 33 | 0.028 | 1.911 | Right Middle Frontal Gyrus 9 |
| 2 | 39 | -5 | 38 | 0.024 | 1.977 | Right Precentral Gyrus 6 |
| 2 | 44 | 0 | 40 | 0.027 | 1.927 | Right Middle Frontal Gyrus 6 |
| 2 | 48 | -2 | 46 | 0.028 | 1.911 | Right Precentral Gyrus 6 |
| 2 | 32 | -6 | 40 | 0.029 | 1.896 | Right Middle Frontal Gyrus 6 |
| 2 | 44 | 2 | 33 | 0.034 | 1.825 | Right Inferior Frontal Gyrus 6 |
| 3 | 16 | 44 | 33 | 0.038 | 1.774 | Right Superior Frontal Gyrus 9 |
| 3 | 22 | 46 | 28 | 0.018 | 2.097 | Right Superior Frontal Gyrus 9 |
| 3 | 14 | 47 | 34 | 1 | 0.000 | Right Superior Frontal Gyrus 8 |
| 3 | 14 | 46 | 34 | 0.023 | 1.995 | Right Superior Frontal Gyrus 8 |
| 3 | 35 | 50 | 24 | 1 | 0.000 | Right Superior Frontal Gyrus 10 |
| 3 | 36 | 41 | 30 | 0.025 | 1.960 | Right Middle Frontal Gyrus 9 |
| 3 | 28 | 40 | 30 | 0.028 | 1.911 | Right Superior Frontal Gyrus 9 |
| 3 | 36 | 34 | 32 | 0.034 | 1.825 | Right Superior Frontal Gyrus 9 |
| 3 | 4 | 52 | 36 | 0.036 | 1.799 | Right Medial Frontal Gyrus 9 |
| 4 | 6 | 0 | 52 | 0.025 | 1.960 | Right Medial Frontal Gyrus 6 |
| 4 | 6.8 | 3.2 | 56.8 | 0.027 | 1.927 | Right Medial Frontal Gyrus 6 |
| 4 | 2 | 2 | 52 | 0.03 | 1.881 | Right Medial Frontal Gyrus 6 |
| 4 | 10 | -2 | 46 | 0.037 | 1.787 | Right Cingulate Gyrus 24 |
| 5 | -44 | -68 | 4 | 0.038 | 1.774 | Left Middle Occipital Gyrus 37 |
| 5 | -44 | -62 | 0 | 0.039 | 1.762 | Left Middle Temporal Gyrus 37 |
| 5 | -48 | -60.8 | -4 | 0.043 | 1.717 | Left Middle Temporal Gyrus 37 |
| 6 | 18 | 14 | 0 | 0.031 | 1.866 | Right Putamen |
| 6 | 20 | 13 | -6 | 0.041 | 1.739 | Right Putamen |

**Table S3: ALE meta-analysis found decreased activities in patients in the following brain regions (consistent across disorders)**

|  | **ADHD** | **ASD** | **BPD** | **MDD** | **OCD** | **SCZ** |
| --- | --- | --- | --- | --- | --- | --- |
| **Contrasts** | 176 | 139 | 151 | 127 | 125 | 130 |
| **Foci** | 1635 | 1462 | 1533 | 1438 | 1433 | 1552 |
| **Subjects** | 3718 | 2602 | 2704 | 344 | 2232 | 2388 |
| **Left hemisphere:** | Cingulate Gyrus  Fusiform Gyrus  Inferior Frontal Gyrus  Inferior Occipital Gyrus  Inferior Parietal Lobule  Insula  Putamen  Medial Frontal Gyrus  Middle Temporal Gyrus  Amygdala  Precentral Gyrus  Precuneus  Superior Frontal Gyrus | Cingulate Gyrus  Inferior Frontal Gyrus  Inferior Occipital Gyrus  Inferior Parietal Lobule  Inferior Temporal Gyrus  Insula  Putamen  Medial Frontal Gyrus  Middle Occipital Gyrus  Middle Temporal Gyrus  Postcentral Gyrus  Precentral Gyrus  Precuneus  Superior Frontal Gyrus  Superior Parietal Lobule  Superior Temporal Gyrus  Thalamus  Transverse Temporal Gyrus | Cingulate Gyrus  Fusiform Gyrus  Inferior Frontal Gyrus  Inferior Parietal Lobule  Inferior Temporal Gyrus  Insula  Medial Frontal Gyrus  Middle Temporal Gyrus  Postcentral Gyrus  Precentral Gyrus  Superior Frontal Gyrus  Superior Temporal Gyrus  Supramarginal Gyrus | Caudate Body  Cingulate Gyrus  Cuneus  Fusiform Gyrus  Inferior Frontal Gyrus  Insula  Putamen  Medial Frontal Gyrus  Precuneus  Superior Frontal Gyrus | Middle Occipital Gyrus  Middle Temporal Gyrus | Inferior Frontal Gyrus  Insula  Middle Frontal Gyrus  Precentral Gyrus  Superior Frontal Gyrus  Thalamus |
| **Right hemisphere:** | Angular Gyrus  Cingulate Gyrus  Inferior Frontal Gyrus  Inferior Parietal Lobule  Insula  Middle Frontal Gyrus  Middle Occipital Gyrus  Middle Temporal Gyrus  Parahippocampal Gyrus  Posterior Cingulate  Precentral Gyrus  Precuneus  Superior Temporal Gyrus  Supramarginal Gyrus  Thalamus | Caudate Body  Cingulate Gyrus  Inferior Parietal Lobule  Insula  Lateral Globus Pallidus  Middle Frontal Gyrus  Parahippocampal Gyrus  Posterior Cingulate  Precentral Gyrus  Precuneus  Superior Frontal Gyrus  Superior Parietal Lobule  Superior Temporal Gyrus  Thalamus | Caudate Body  Cingulate Gyrus  Fusiform Gyrus  Inferior Parietal Lobule  Insula  Middle Frontal Gyrus  Middle Occipital Gyrus  Middle Temporal Gyrus  Precentral Gyrus  Superior Frontal Gyrus  Superior Parietal Lobule  Superior Temporal Gyrus  Supramarginal Gyrus  Thalamus | Angular Gyrus  Cingulate Gyrus  Fusiform Gyrus  Inferior Frontal Gyrus  Inferior Parietal Lobule  Medial Frontal Gyrus  Posterior Cingulate  Precentral Gyrus  Precuneus  Superior Frontal Gyrus  Superior Parietal Lobule  Thalamus | Angular Gyrus  Cingulate Gyrus  Inferior Frontal Gyrus  Inferior Parietal Lobule  Putamen  Medial Frontal Gyrus  Middle Temporal Gyrus  Precentral Gyrus  Precuneus  Superior Frontal Gyrus  Superior Parietal Lobule  Superior Temporal Gyrus  Thalamus | Angular Gyrus  Cingulate Gyrus  Claustrum  Fusiform Gyrus  Inferior Frontal Gyrus  Inferior Occipital Gyrus  Insula  Lingual Gyrus  Medial Frontal Gyrus  Precentral Gyrus  Superior Frontal Gyrus  Superior Parietal Lobule  Thalamus |

**Table S4: ALE meta-analysis found increased activities in patients in the following brain regions (not consistent across disorders)**

|  | **ADHD** | **ASD** | **BPD** | **MDD** | **OCD** | **SCZ** |
| --- | --- | --- | --- | --- | --- | --- |
| **Contrasts** | 176 | 139 | 151 | 127 | 125 | 130 |
| **Foci** | 1635 | 1462 | 1533 | 1438 | 1433 | 1552 |
| **Subjects** | 3718 | 2602 | 2704 | 344 | 2232 | 2388 |
| **Left hemisphere:** | Medial Frontal Gyrus | Claustrum  Insula  Medial Frontal Gyrus  Subcallosal Gyrus | Caudate Body  Cingulate Gyrus  Insula  Lateral Globus Pallidus  Middle Frontal Gyrus  Middle Temporal Gyrus  Precuneus  Superior Frontal Gyrus | Caudate Body  Inferior Frontal Gyrus  Medial Frontal Gyrus  Middle Temporal Gyrus  Postcentral Gyrus  Precentral Gyrus  Superior Temporal Gyrus  Transverse Temporal Gyrus | Caudate Body  Inferior Frontal Gyrus | Angular Gyrus  Anterior Cingulate  Cuneus  Inferior Parietal Lobule  Middle Frontal Gyrus  Postcentral Gyrus  Precentral Gyrus  Precuneus  Superior Parietal Lobule  Supramarginal Gyrus |
| **Right hemisphere:** | N/A | Anterior Cingulate | Middle Frontal Gyrus  Posterior Cingulate  Precuneus  Superior Temporal Gyrus  Supramarginal Gyrus | Anterior Cingulate  Inferior Frontal Gyrus  Inferior Temporal Gyrus  Medial Frontal Gyrus  Middle Frontal Gyrus  Middle Temporal Gyrus  Superior Temporal Gyrus  Transverse Temporal Gyrus | Anterior Cingulate  Cingulate Gyrus  Posterior Cingulate  Thalamus | Medial Frontal Gyrus  Postcentral Gyrus  Precentral Gyrus  Precuneus |

**Table S5: The factor scores of brain regions in factor analyses**

| **ROI** | **Factor 1** | **Factor 2** | **Factor 3** | **Factor 4** |
| --- | --- | --- | --- | --- |
| **R Insula** | -2.115 | -0.311 | 0.099 | 0.079 |
| **R Inferior Frontal Gyrus** | 0.301 | 1.747 | -0.102 | 0.601 |
| **R Precentral Gyrus** | -1.15 | 1.596 | -1.936 | -0.122 |
| **R Thalamus** | 2.334 | -0.288 | -0.217 | 0.265 |
| **L Inferior Frontal Gyrus** | 0.116 | -2.973 | -0.456 | -0.068 |
| **L Precentral Gyrus** | 0.267 | 1.488 | -1.905 | -0.24 |
| **L Middle Frontal Gyrus** | 0.976 | -0.674 | -0.848 | -0.079 |
| **L Insula** | 1.618 | 0.579 | -0.27 | 0.293 |
| **R Superior Parietal Lobule** | -1.872 | -0.628 | 0.363 | 0.331 |
| **R Angular Gyrus** | -0.434 | 0.241 | -0.126 | 1.92 |
| **L Superior Frontal Gyrus** | -0.028 | -0.984 | -0.901 | -0.1 |
| **R Medial Frontal Gyrus** | -0.912 | 0.762 | 0.299 | -0.183 |
| **R Cingulate Gyrus** | -0.173 | -0.378 | -0.696 | -0.784 |
| **R Superior Frontal Gyrus** | 0.832 | -0.04 | 0.059 | -3.108 |
| **R Fusiform Gyrus** | 0.411 | -0.601 | -0.127 | -0.047 |
| **L Cingulate Gyrus** | -0.052 | -0.366 | -0.612 | -0.024 |
| **R Precuneus** | 0.359 | 0.784 | -0.161 | -1.46 |
| **R Inferior Parietal Lobule** | 1.194 | -0.352 | -0.02 | 1.875 |
| **R** **Middle Temporal Gyrus** | 0.148 | 0.022 | -0.562 | 2.301 |
| **L Putamen** | -1.064 | 1.451 | 0.391 | -0.062 |
| **R Middle Frontal Gyrus** | -0.184 | -0.844 | 0.534 | -0.187 |
| **R Posterior Cingulate** | 0.412 | 0.527 | 1.384 | -0.059 |
| **L** **Inferior Parietal Lobule** | 1.041 | 0.379 | 2.025 | 0.07 |
| **L** **Medial Frontal Gyrus** | 0.61 | 0.658 | 2.277 | -0.007 |
| **R Superior Temporal Gyrus** | -1.094 | 0.179 | 1.974 | -0.042 |
| **L Middle Temporal Gyrus** | -0.336 | -0.148 | -0.334 | -1.133 |
| **L Precuneus** | -0.294 | -0.363 | -0.629 | 0.002 |

**Table S6：Regression analysis on the original scores of SCZ and ASD with the factor 1 scores**

|  |  | **SCZ** | |  |  |  | **ASD** | |  |
| --- | --- | --- | --- | --- | --- | --- | --- | --- | --- |
|  | **Res.** | **Std.**  **Res.** | **Pred.** | **Orig.** |  | **Res.** | **Std.**  **Res.** | **Pred.** | **Orig.** |
| **R Insula** | 0.062 | 0.368 | 2.668 | 2.73 |  | 0.108 | 0.36 | 2.122 | 2.23 |
| **R Precentral Gyrus** | -0.116 | -0.692 | 2.486 | 2.37 |  | -0.213 | -0.71 | 2.323 | 2.11 |
| **L Thalamus** | -0.28 | -1.668 | 1.34 | 1.06 |  | -0.49 | -1.636 | 3.24 | 2.75 |
| **R Thalamus** | 0.203 | 1.206 | 1.647 | 1.85 |  | 0.368 | 1.23 | 2.922 | 3.29 |
| **L Inferior Frontal Gyrus** | 0.009 | 0.052 | 2.041 | 2.05 |  | 0.01 | 0.035 | 2.45 | 2.46 |
| **L Precentral Gyrus** | 0.116 | 0.687 | 2.144 | 2.26 |  | 0.232 | 0.774 | 2.598 | 2.83 |
| **L Middle Frontal Gyrus** | -0.003 | -0.021 | 1.943 | 1.94 |  | 0.003 | 0.01 | 2.677 | 2.68 |
| **L Insula** | 0.125 | 0.742 | 1.855 | 1.98 |  | 0.195 | 0.65 | 2.825 | 3.02 |
| **R Superior Parietal Lobule** | -0.155 | -0.925 | 2.625 | 2.47 |  | -0.248 | -0.829 | 2.118 | 1.87 |
| **L Superior Frontal Gyrus** | 0.059 | 0.35 | 2.141 | 2.2 |  | 0.111 | 0.369 | 2.479 | 2.59 |
| **R Cingulate Gyrus** | -0.053 | -0.314 | 2.243 | 2.19 |  | -0.154 | -0.514 | 2.564 | 2.41 |
| **R Superior Frontal Gyrus** | 0.036 | 0.214 | 2.124 | 2.16 |  | 0.078 | 0.261 | 3.032 | 3.11 |

* Predicted scores (Pred.);

Corresponding residual (Res.);

Standardized residual (Std. Res.);

Original scores (Orig.)
